# Strain tunable quantum emission from atomic defects in hexagonal boron nitride for telecom-bands

**DOI:** 10.1038/s41598-022-26061-w

**Published:** 2022-12-15

**Authors:** Akbar Basha Dhu-al Shaik, Penchalaiah Palla

**Affiliations:** Department of Micro and Nanoelectronics, School of Electronics Engineering, Vellore Institute of Technology, Vellore, Tamil Nadu 632014 India

**Keywords:** Single photons and quantum effects, Nanophotonics and plasmonics, Quantum optics

## Abstract

This study presents extending the tunability of 2D hBN Quantum emitters towards telecom (C-band − 1530 to 1560 nm) and UV-C (solar blind − 100 to 280 nm) optical bands using external strain inducements, for long- and short-range quantum communication (Quantum key distribution (QKD)) applications, respectively. Quantum emitters are the basic building blocks of this QKD (quantum communication or information) technologies, which need to emit single photons over room temperature and capable of tuning the emission wavelength to the above necessary range. Recent literature revealed that quantum emitters in 2D hBN only has the ability to withstand at elevated temperatures and aggressive annealing treatments, but density functional theory (DFT) predictions stated that hBN can only emit the single photons from around 290 to 900 nm (UV to near-IR regions) range. So, there is a need to engineer and further tune the emission wavelength of hBN quantum emitters to the above said bands (necessary for efficient QKD implementation). One of the solutions to tune the emission wavelength is by inducing external strain. In this work, we examine the tunability of quantum emission in hBN with point defects by inducing three different normal strains using DFT computations. We obtained the tunability range up to 255 nm and 1589.5 nm, for the point defects viz boron mono vacancies (V_B_) and boron mono vacancies with oxygen atoms (V_B_O_2_) respectively, which can enhance the successful implementation of the efficient QKD. We also examine the tunability of the other defects viz. nitrogen mono vacancies, nitrogen mono vacancy with self-interstitials, nitrogen mono vacancy with carbon interstitials, carbon dimers and boron dangling bonds, which revealed the tunable quantum emission in the visible, other UV and IR spectrum ranges and such customized quantum emission can enhance the birth of other quantum photonic devices.

## Introduction

Photo-luminescent quantum emitters which are close enough to ideal single photon emission characteristics, capable of holding the emissive properties at higher operating temperatures, various harsh environments and possibility to tune the emission spectrum to the wide range (higher to shorter wavelength range) are the central elements for implementing successful quantum information technologies and integrated quantum photonics. In particular, robust quantum communications demand quantum emitters that provide efficient quantum emission in the telecom (C-band) range of 1530–1560 nm for longer and short-range distances^1–3^ via optical fibers^4^ and free-space channels^5,6^. Quantum communication in UV-region is also another alternative approach to short-range distances [under non-line of sight (NLOS) condition], which requires quantum emission in solar-blind (UV-C) region of 100–280 nm range^7,8^.

The state-of-the-art research revealed that, implementing such ideal quantum emitters using layered materials is one of the most promising solutions^9–12^. However, to-date quantum emitters developed in 2D hBN (white graphene) are found to sustain their high emission characteristics at elevated operating temperatures^13^ and vigorous annealing treatments^14^, but exhibits the emission spectrum only from UV to near-IR region i.e., around 290–900 nm range^15,16^. As an alternative technique, carbon nanotubes exhibit the quantum emission around 1500 nm^17^, but faces the drawback of narrow emission range and their low operating temperatures. On the other hand, quantum dots can achieve broad emission spectrum^18,19^. However, the specific wavelength emission in quantum dots requires distinct quantum arrangements and dissimilar doping. Hence, it is difficult to achieve complete wide-range emission spectrum on a single host material using quantum dots.

One of the most promising solutions to fulfil the quantum communication requirement is by tuning the quantum emission in 2D hBN through defects to the necessary range. As hBN is the first known natural hyperbolic material, i.e., in-plane bonds are stronger than out-of-plane bonds in its crystal structure, external strain can be applied to modify the electronic energy levels of luminescent point defect states and tune their emission spectrum. The high stretchability feature^20^ of 2D materials supports to strain engineer the electronic band gap of quantum emitters and promotes the tunability of single photon emission. Here, we illustrate the optical tunability of precise quantum emitters (Luminescent point defects) in 2D hBN by inducing strain gradients, using density functional theory (DFT) calculations.

**Figure 1:**
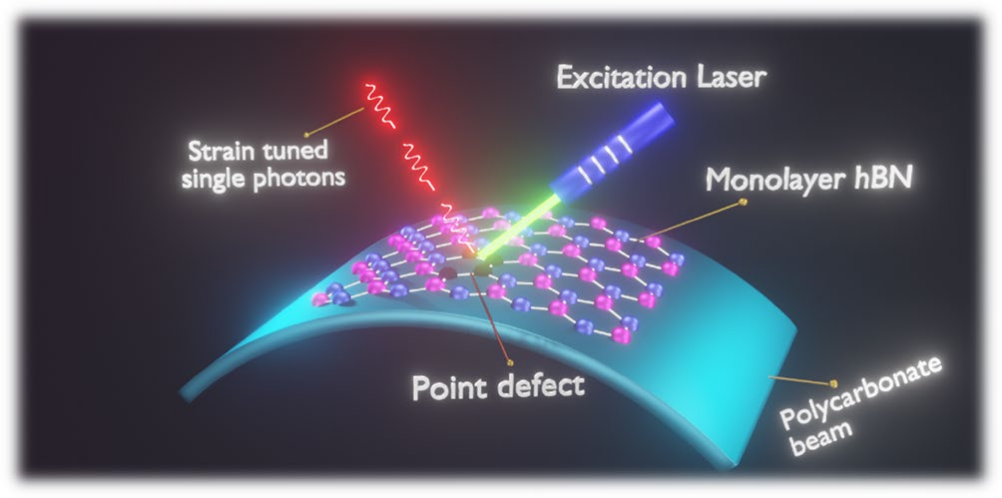
Schematic illustration of quantum emission tuning towards near-IR region by external strain inducement. The luminescent point defect in hBN as formed by various defect fabrication processes, was subjected to external strain using bendable polycarbonate beam, under optical laser excitation to tune the quantum emission wavelength to the telecom wavelength range in order to meet the quantum communication requirement.

In this work, we simulated three different types of normal strain inducements in 2D hBN with point defects by approximating an experimental situation such as inducing strain on 2D hBN layer with point defects using a bendable polycarbonate (PCB) substrate^21^. In order to identify potential quantum emission candidates (luminescent point defects), to employ for our strain inducements simulations, earlier published DFT simulations without strain inducements has taken into consideration^22–24^. Our DFT simulated results of quantum emitters without external strain inducement also consistent with the earlier published experimental observations. From our work with various types of strain inducements, it is observed that the emitters exhibit greater tunability and the broad emission spectrum from solar blind region (UV-C) of 255 nm to beyond telecom wavelength range of 1589.5 nm, respectively. The schematic illustration of tuned near-IR single photon emission from the quantum emitters subjected to external strain under optical excitation is shown in Fig. 1.

## Materials and methodology

**Figure 2:**
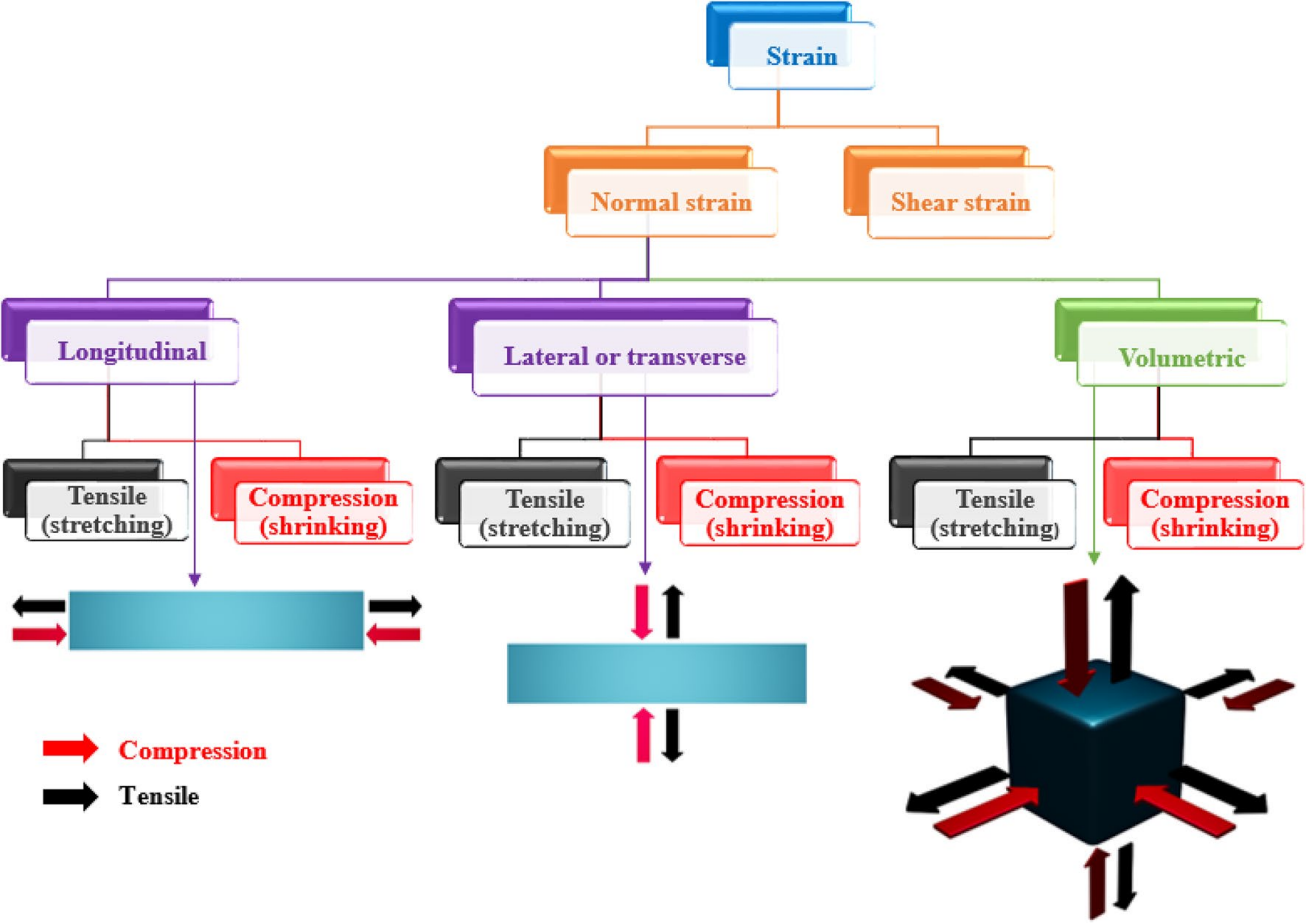
Classification of different externally inducible strains and their schematics. Overall, the externally inducible strain is classified into two types, in which we concern on inducing different normal strains. This normal strain is classified into three types: longitudinal (applying strain in horizontal direction w.r.t the material), lateral (applying strain in vertical direction w.r.t the material) and volumetric (applying strain through all the sides) and best examples of volumetric strain is biaxial strain. The schematic representation of three different normal strain inducements were shown below. In all the three different normal strains, there are only two possible ways of creating strain effects, that is creating either tensile or compression effects. Tensile effect creates stretching of material and compression creates shrinking of material. For example: longitudinal tensile strain is stretching the material towards horizontal direction and longitudinal compressive strain is shrinking of material in horizontal direction and as similarly lateral and volumetric tensile and compressive strain effects behaves.

In this article, we majorly concentrate to surveil the broader tunability of quantum emitters in 2D hBN under different normal strains induced. Figure 2 represents the classification of various externally inducible strains. We mainly focus on different normal strain induced tunabilities, which were observed in earlier strain inducing experiments^14,25^ and computational works^14,21^ and here we projected the emission tunability of different luminescent point defects for different normal strains induced. Inducing shear strain is out of our research interest.

**Figure 3:**
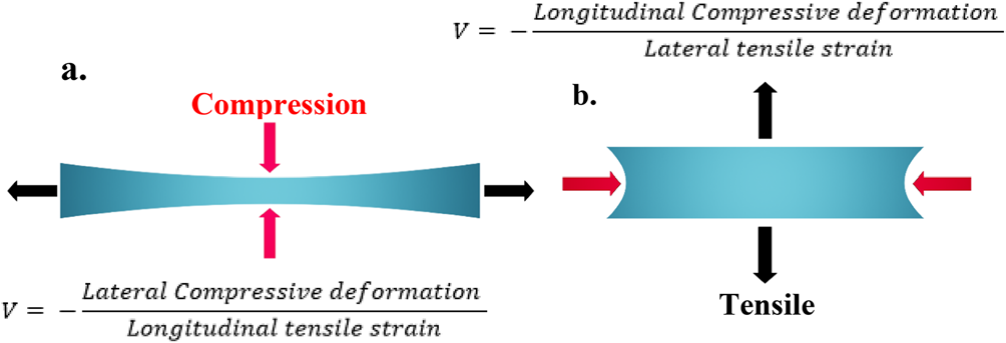
Schematic representation of Poisson’s ratio effect for the induced longitudinal and lateral strains. (**a**) For the applied longitudinal tensile strain and then along with longitudinal stretching (horizontal stretching), there will also be a small compressive deformation along orthogonal direction due to Poisson’s ratio (*V*) effect of material and it is defined by the expression below (**a**). (**b**) For the applied lateral tensile strain and then along with lateral stretching (vertical stretching), there will also be a small compressive deformation along orthogonal direction due to Poisson’s ratio (*V*) effect of material and it is defined by the expression above (**b**). Here, in both the figures, we induced tensile (stretching) strain, and due to that the compressive deformation takes place. The deformation due to Poisson’s ratio (*V*) can also be a small stretching (tensile), when actual induced strain is compressive.

One important phenomenon needs to be considered while computing this strain inducements, particularly, while inducing transverse or longitudinal strains is the Poisson’s ratio. The Poisson’s ratio (*V*) defines that along with strain induced (transverse or longitudinal), there will also be another small deformation of the material, which is perpendicular to the load direction (applied strain direction). This small deformation will be in negative ratio as shown in Fig. 3a,b.

As per earlier literature (strain induced quantum tuning), we found three major types of normal strains, studied in references^21,25^. One is biaxial strain (volumetric strain) and the other two are one-sided lateral and one-sided longitudinal strains. This kind of one-sided lateral or longitudinal strain can be applied to the material, by placing it on a bendable polycarbonate (PC) beam (in which one edge of either vertical or horizontal direction is fixed and strain is applied at another edge) as observed in reference^21^. Hence, by computing these three strain types, we examine the quantum emission tunability of different luminescent point defects in 2D hBN and project their tunability.

### Inducement of biaxial strains

**Figure 4:**
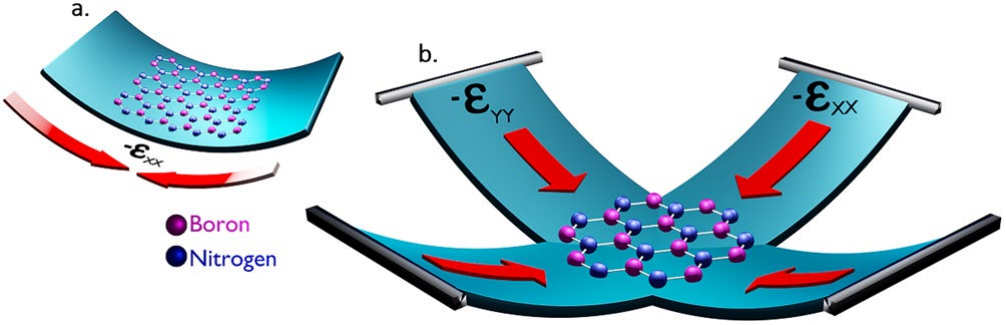
Schematic illustration of negative biaxial strain inducement to hBN film. The hBN film is transferred to PC beam arranged in cruciform structure to induce biaxial strain. All the edges are bent upwards to induce negative biaxial strain which leads to overall shrinking of material. (**a**) Shrinking (compressive) force induced towards only tensor xx component will leads to negative uniaxial strain. (**b**) As similar to (**a**), if compressive force is induced in both tensor xx and yy components then it will lead to negative biaxial strain.

**Figure 5:**
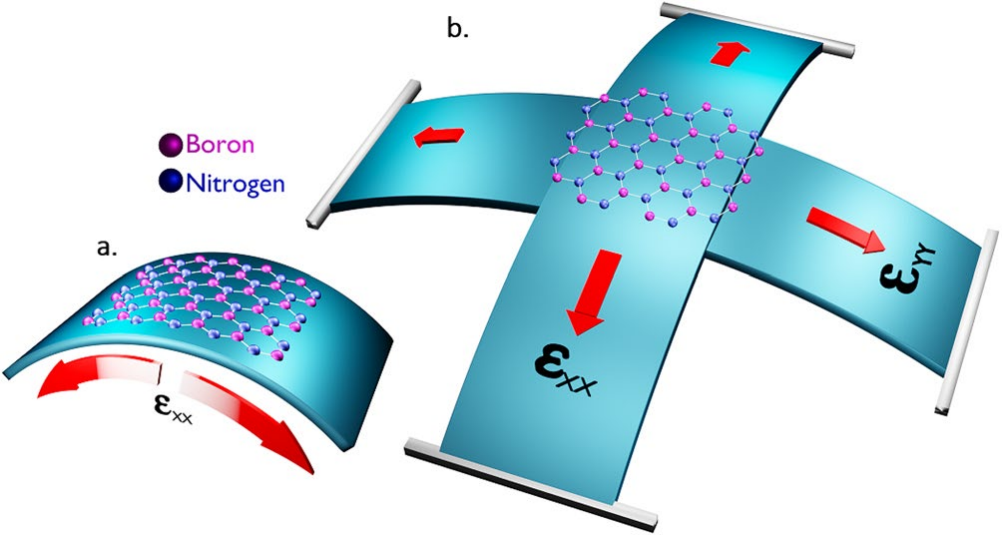
Schematic illustration of positive biaxial strain inducement to hBN film. The hBN film is transferred to PC beam arranged in cruciform structure to induce biaxial strain. All the edges are bent downwards to induce positive biaxial strain which leads to overall stretching of material. (**a**) Expansive (tensile) force induced towards only tensor xx component will leads to positive uniaxial strain. (**b**) As similar to (**a**), if expansive force is induced in both tensor xx and yy components then it will lead to positive biaxial strain.

We computed the effect of biaxial strain to the hBN quantum emitters using DFT simulations by considering experimental conditions. In practise, a negative (positive) biaxial strain can be applied by depositing the hBN film on the core of PC beams, arranged in cruciform structure as observed in Ref.^26^ and bending all the edges upwards (downwards) simultaneously, as shown in Fig. 4 (Fig. 5), induces the complete shrinking (expansive) forces in both xx and yy tensor directions to the material at same instance of time.

By using target-stress tensor section present in geometrical optimizer in DFT computations, we simulated the effect of negative (positive) biaxial strains to various luminescent point defects in 2D hBN. All the stress error tolerance values were set to 0.0005 eV/A^3^.

The target-stress tensor section, targets the internal stress of the material, where negative strain values along xx and yy components results in compression force along the xx and yy (tensor) directions of 2D hBN as shown in Fig. 4, which leads to negative biaxial strain inducement and positive values along xx and yy components will results in expansion force along the xx and yy (tensor) directions of 2D hBN as shown in Fig. 5, which leads to positive biaxial strain inducement.

### Inducement of one-sided lateral and one-sided longitudinal strains

**Figure 6:**
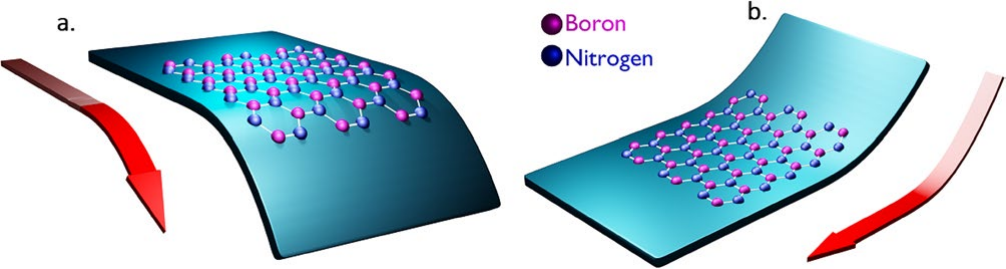
Schematic illustration of tensile and compressive effects. The hBN film transferred to bendable polycarbonate beam and one edge of the beam is fixed. (**a**) Another edge of PC beam is bent downwards to induce tensile strain, which leads to stretching of material towards tensile strain induced edge. (**b**) Another edge of PC is bent upwards to induce compressive strain, which leads to shrinking of material towards compressive strain induced edge.

We also computed the effect of one-sided lateral and one-sided longitudinal strains to hBN quantum emitters using DFT simulations, to examine the quantum tunability. This approach of inducing constrained normal strains (one-sided lateral or one-sided longitudinal strain) in order to efficiently tune the quantum emission, was considered from earlier experimental and DFT observations as shown in reference^21^. This kind of normal strain (one-sided lateral or one-sided longitudinal strain) was induced experimentally, by transferring the hBN film on to a 1.5-mm thick bendable polycarbonate (PC) beam, which allows to induce controllable strain. One edge (either vertical or horizontal) of the PC beam will be fixed and the other side will be bent downward (upward) to produce tensile (compressive) effects as shown in Fig. 6a,b.

While simulating this kind of one-sided lateral or one-sided longitudinal strains inducement as similar to experimental approximation, the Poisson’s ratio effect as shown in Fig. 3, also comes into consideration.

According to Poisson’s ratio, when a tensile (stretching) effect is induced towards one-sided lateral (vertical) [or longitudinal (horizontal)] direction, it creates an expansion of material (towards one-sided lateral or longitudinal direction) and also produces a small compressive deformation. This compressive deformation will be orthogonal to applied strain (tensile effect) direction.

Similarly, when a compression (shrinking) effect is induced towards one-sided lateral (vertical) [or longitudinal (horizontal)] direction, it creates shrinking of material (towards one-sided lateral or longitudinal direction) and also produces a small expansive deformation. This expansive deformation will be orthogonal to applied strain (compressive effect) direction.

**Figure 7:**
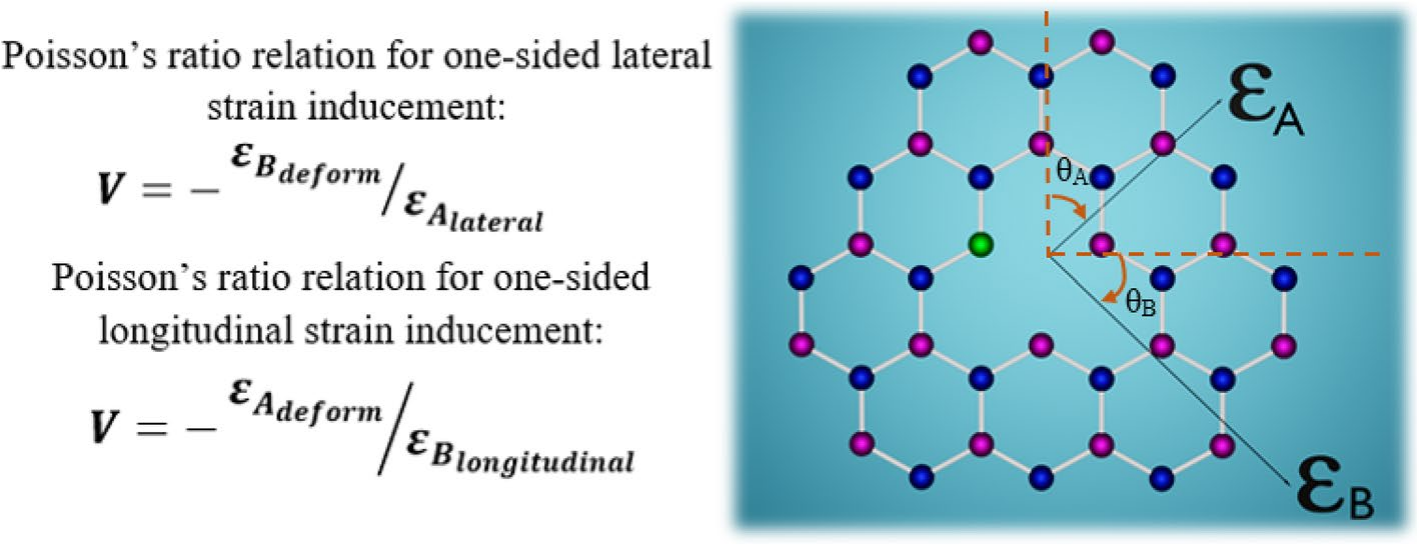
Poisson’s ratio relations and corresponding orthogonal strain directions. According to Poisson’s ratio, as shown in Fig. 3, when tensile (stretching) effect is induced in both sides of lateral (vertical) direction, then the compressive (shrinking) deformation takes place in both the sides longitudinal (horizontal) direction (which is orthogonal) and vice-versa. But, for one-sided lateral or longitudinal strain inducement, the strain is induced in one edge of vertical or horizontal directions respectively, by fixing another edge and so that the deformation due to Poisson’s ratio will also be only one side (which can be orthogonal). Now, for the 2D hBN with point defect (C_B_V_N_), inducing one-sided lateral strain means inducing strain towards Ɛ_A_ direction (whose direction is almost vertical, with a small tilt of θ_A_) and due to Poisson’s ratio, along with actual lateral strain, a small deformation will also occur at orthogonal position i.e., Ɛ_B_ direction (whose direction is orthogonal to Ɛ_A_). The amount of deformation can be known by the expression (Poisson’s ratio relation for one-sided lateral strain inducement). This Poisson’s ratio is always negative (If the one-sided lateral strain towards Ɛ_A_ is tensile then the deformation due to Poisson’s ratio towards Ɛ_B_ is compressive and vice versa if the actual strain is compressive and then the small deformation is tensile). Similarly, inducing one-sided longitudinal strain means inducing strain towards Ɛ_B_ direction (whose direction is almost horizontal, with a small tilt of θ_B_) and due to Poisson’s ratio, along with actual longitudinal strain, a small deformation will also occur at orthogonal position i.e., Ɛ_A_ direction (whose direction is orthogonal to Ɛ_B_). The amount of deformation can be known by the expression (Poisson’s ratio relation for one-sided longitudinal strain inducement). If this one-sided longitudinal strain towards Ɛ_B_ is tensile then the deformation due to Poisson’s ratio towards Ɛ_A_ is compressive and vice versa if the actual strain is compressive and then the small deformation is tensile. Important point to be noted is these small tilts of θ_A_ and θ_B_ from the mean positions was observed and considered from earlier strain inducing DFT computations as shown in Ref.^21^.

Hence, to compute these effects in a realistic way, we considered the Poisson’s ratio of polycarbonate (PC) beam = 0.37, by which strain is induced to 2D hBN quantum emitters as observed in reference^21^. The Poisson’s ratio relation for inducing one-sided lateral and one-sided longitudinal strain to the hBN point defects, using a bendable polycarbonate (PC) beam is represented in Fig. 7. The two orthogonal strain directions (A and B) as shown in Fig. 7, were assigned along the plane of defective hBN film in order to simulate this kind of constrained normal strains (one-sided lateral and one-sided longitudinal strains).

This strategy of assigning these two orthogonal strain directions, to compute one-sided lateral and one-sided longitudinal strain inducement to the point defects in 2D hBN and considering experimental approximations such as Poisson’s ratio of PC beam were acquired from earlier DFT computations as in reference^21^.

We simulated these two orthogonal strain inducements (due to Poisson’s effect) in mutually exclusive method with the help of atomic constraint editor using geometry optimizer in DFT computations, by setting the stress error tolerance to 0.0005 eV/A^3^.

### Parameters considered and plane wave versus Gaussian basis set calculations

Initially, a few DFT computations for performing volumetric strain inducements, like biaxial strains, were carried out for the luminescent point defect (C_B_V_N_), using plane-wave calculations.

All plane-wave calculations are spin-polarized. A plane-wave cut-off of 450 eV was used for the calculations and by using Generalized Gradient Approximation (GGA) to the exchange correlation functional proposed by Perdew, Burke and Ernzerhof (PBE)^27^. The nucleus–electron interaction is represented by projector augmented wave (PAW) pseudopotentials. A Gaussian smearing occupation is employed for numerical accuracy, with a broadening of 0.05 eV and to an energy tolerance of 0.01 eV.

Pristine single-layer hBN was first geometry-optimized using a 21 × 21 × 1 Monkhurst–Pack reciprocal space grid. The defective hBN monolayer was created using a 7 × 7 supercell and the defective structures were re-optimized with the reciprocal space grid reduced to 3 × 3 × 1.

Next, we re-performed the same biaxial strain inducement simulations to the same luminescent point defect (C_B_V_N_), by employing Linear combination of atomic orbitals (LCAO) calculations. In LCAO, all the parameters and approximations were similar to the above plane-wave calculations and the only difference is projector augmented wave (PAW) pseudopotentials is replaced with Fritz-Haber Institute (FHI) pseudopotentials, which computed according to the procedure reported by Troullier and Martins^28^ and the Double-zeta plus polarization (DZP) basis set was employed for the calculations.

**Figure 8:**
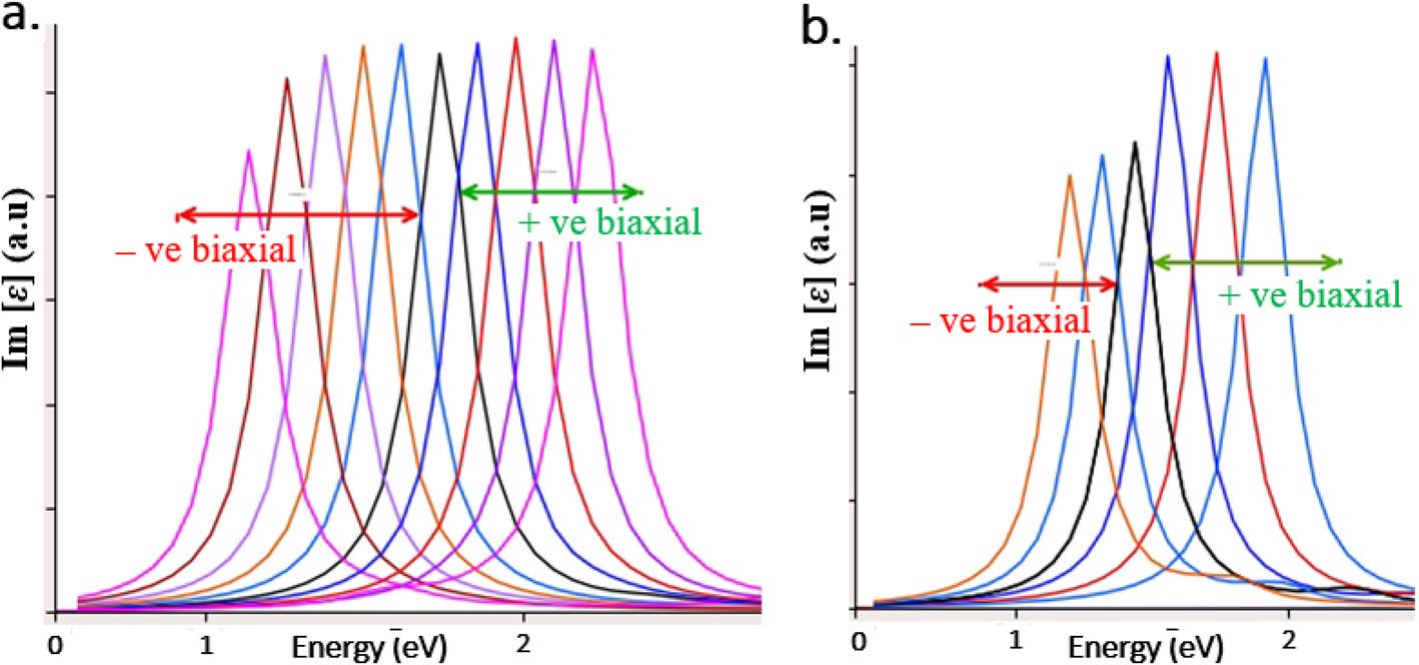
Biaxial strain inducement simulations using plane-wave and LCAO calculations, for C_B_V_N_ defect. (**a**) + ve and −ve biaxial strain inducement simulation of C_B_V_N_ defect using plane-wave calculations. (**b**) + ve and −ve biaxial strain inducement simulation of C_B_V_N_ defect using LCAO calculations. Both the plane-wave and LCAO calculations exhibit similar tuning of quantum emission from C_B_V_N_ defect towards lower and higher energy regions. We computed ⁓ 3.45% positive and negative biaxial strain (– 3.45% to + 3.45%). The black colour peak in both the figures (**a**,**b**) indicates ZPL quantum emission at 1.7 eV from C_B_V_N_ defect under no external strain (zero strain) condition. For + ve biaxial strain tunability is observed upto 2.05 eV (blue-shifted) and for −ve biaxial strain tunability is observed upto 1.22 eV (red-shifted).

By comparing the biaxial strain inducement simulations of the point defect (C_B_V_N_), using plane-wave and LCAO calculations, we observed no major difference in the obtained optical spectrum results as shown in Fig. 8. Figure 8a,b represents biaxial strain induced tuning of quantum emission from C_B_V_N_ defect, using plane-wave and LCAO calculations, respectively.

For the simulation results shown in Fig. 8, we computed the biaxial strain of ⁓ 3.45%. However, the mechanical properties of monolayer boron nitride experimentally revealed that, it has the fracture strength of 70.5 ± 5.5 GPa^29^, which can tolerate the maximum strain upto ⁓ 8.15%.

Hence, we observed that the results of strain tuning with plane-wave and LCAO calculations were almost consistent with each other and so that we performed rest of the biaxial, one-sided lateral and one-sided longitudinal strain inducement simulations to other luminescent point defects with LCAO calculators and as similar, rest of all the defective hBN monolayers was created using a 7 × 7 supercell and was geometrically optimized using Monkhurst-Pack reciprocal space grid to a density around 3 × 3 × 1.

All these DFT computations were performed using Synopsys QuantumATK Q-2019.12-SP1 software package (atomic-scale modelling software)^30^.

The precise luminescent point defects, which we employed for strain inducing simulations were earlier demonstrated (with published experiments) as the reliable single photon emitters as listed in Table 1^31–35^. These defects’ single photon emission with GW approximations (first principle calculations) are beyond the scope of our work.

**Table 1:** Experimental defect fabrication process and corresponding references (Published literature).

Defect name	Fabrication process	References
V_B_O_2_	Plasma etching with a power of 200 W under a pressure of 180 mTorr of Ar or O_2_ for 2 min at room temperature	^37^
C_B_V_N_	Controlled incorporation of impurities (carbon) via efficient bottom-up synthesis methods like MOVPE, MBE and HOPG or controlled carbon ion implantation at an energy of 10 keV	^38^
N_B_V_N_	Annealing at 850 °C under 1 torr of argon for 30 min	^39^
Boron dangling bonds	Boron dangling bonds defect can be formed when the systematic bonding arrangement in a crystalline material is hampered. These are mostly detected at surfaces, interfaces, grain boundaries and in-voids. These defects can be formed with precise ion irradiation with energy of 50–70 keV	^40,41^
C_B_C_N_	Carbon dimer is expected to form when the carbon is involved during growth of hBN films	^42^
V_BN_	Irradiation of high electron beam with energy around 100 keV	^43^
V_B_	Controlled energetic electron beam irradiation (energy 120 keV) through a layer-by-layer sputtering process. Boron mono vacancies are more preferably formed and nitrogen mono vacancies are detected at dominating zig-zag type edges	^44^
V_N_		

## Results and discussion

By performing constrained DFT simulations^36^, initially we cross-examined the optical emission spectrums and the corresponding projected density of states (PDOS) of precise luminescent point defects (V_B_O_2_, C_B_V_N_, N_B_V_N_, boron dangling bonds, C_B_C_N_, V_BN_, V_B_ and V_N_) with earlier published literature^37–44^, without inducing any external strain inducement. We have selected these precise luminescent point defects, based on their consistence of experimental observations with DFT approximations and simple fabrication possibilities as shown in Table 1.

These luminescent point defects create intermediate energy levels (an electron occupied ground state and un-occupied excited state) between valence and conduction bands of 2D hBN as shown in reference^39^. When this occupied ground state electron is excited with enough energy, it transmits to un-occupied excited state. As this ground and excited state transition is based on single electron, it emits a single photon of specific wavelength while relaxing back to ground state. The DFT studies acknowledges this single photon emission by a Lorentzian shape peak (sharp emission peak), which is a signature of quantum emission and this Lorentzian shaped sharp emission peak is also considered as zero-phonon line (ZPL), by quantum studies.

The DFT computed optical emission spectrums of luminescent point defects under no external strain inducement (which were experimentally realized and published as listed in Table 1) exhibits sharp zero-phonon lines (ZPL), in Lorentzian shape at their respective energies as listed in Table 2 and this sharp Lorentzian shaped ZPLs confirmed the quantum emission nature of point defects at their appropriate energies.

**Table 2:** Information related to defect type, defect ZPL and correlated PDOS information (simulated work).

Defect name	Defect schematic no	ZPL energy	PDOS figure no
V_B_O_2_	Figure 9a	1.26 eV	Figure 9b
C_B_V_N_	Figure 11a	1.74 eV	Figures S2a, S3a
N_B_V_N_	Figure 11d	2 eV	Figures S2d, S3d
Boron dangling bonds	Figure 11g	3.18 eV	Figure S3g
C_B_C_N_	Figure 12a	3.54 eV	Figure S4a
V_BN_	Figure 12d	3.5 eV	Figure S4d
V_B_	Figure 10a	4.7 eV	Figure 10b
V_N_	Figure 12g	3.59 eV	Figure S4g

*S1 and S2 indicates supporting information Figs. 1 and 2, respectively.

Also, the DFT computed PDOS, exhibits a graphical representation of intermediate states (electron occupied and un-occupied energy states) formed due to the point defects and energy difference between these occupied and un-occupied states is found to be consistent with ZPL energies of corresponding luminescent point defects, which further strongly confirms the quantum emission signature of point defects. The complete DFT computed information related to various point defects, their schematics, computed ZPL quantum emission energies and corresponding PDOS information are listed in Table 2.

As from the simulated data observed from Table 2, point defects in 2D hBN exhibits the single photon emission from 1.26 eV (980 nm) to 4.7 eV (260 nm). Thus, the quantum emission from 2D hBN may partially support the quantum communication in UV-C (solar blind region) for short distances. But, the quantum communication for long distances requires the quantum emission in telecom (C-band) range of 1530–1560 nm, which may not be possible with near-IR emission of quantum emitters in 2D hBN.

Hence, in order to fulfil the QKD (quantum communication) requirement for long distances and as well as to still enhance the short-range UV communications, we attempted the possible emission tunability of precise quantum emitters by inducing external strain.

### Tunability of quantum emitters in different optical bands by external strain inducement

#### Quantum emission tunability of V_B_O_2_ defect in IR region

The first and foremost consideration while tuning the quantum emission towards IR region is, selecting luminescent point defects whose quantum emission (ZPL) energy is around the frontier of near-IR region, which makes it easier to efficiently tune the quantum emission towards deep IR region. In such a way, among all the luminescent point defects in 2D hBN only V_B_O_2_ defect exhibits emission in near-IR region, whose DFT approximations were found to be consistent with experimental observations as listed in Table 1.

**Figure 9:**
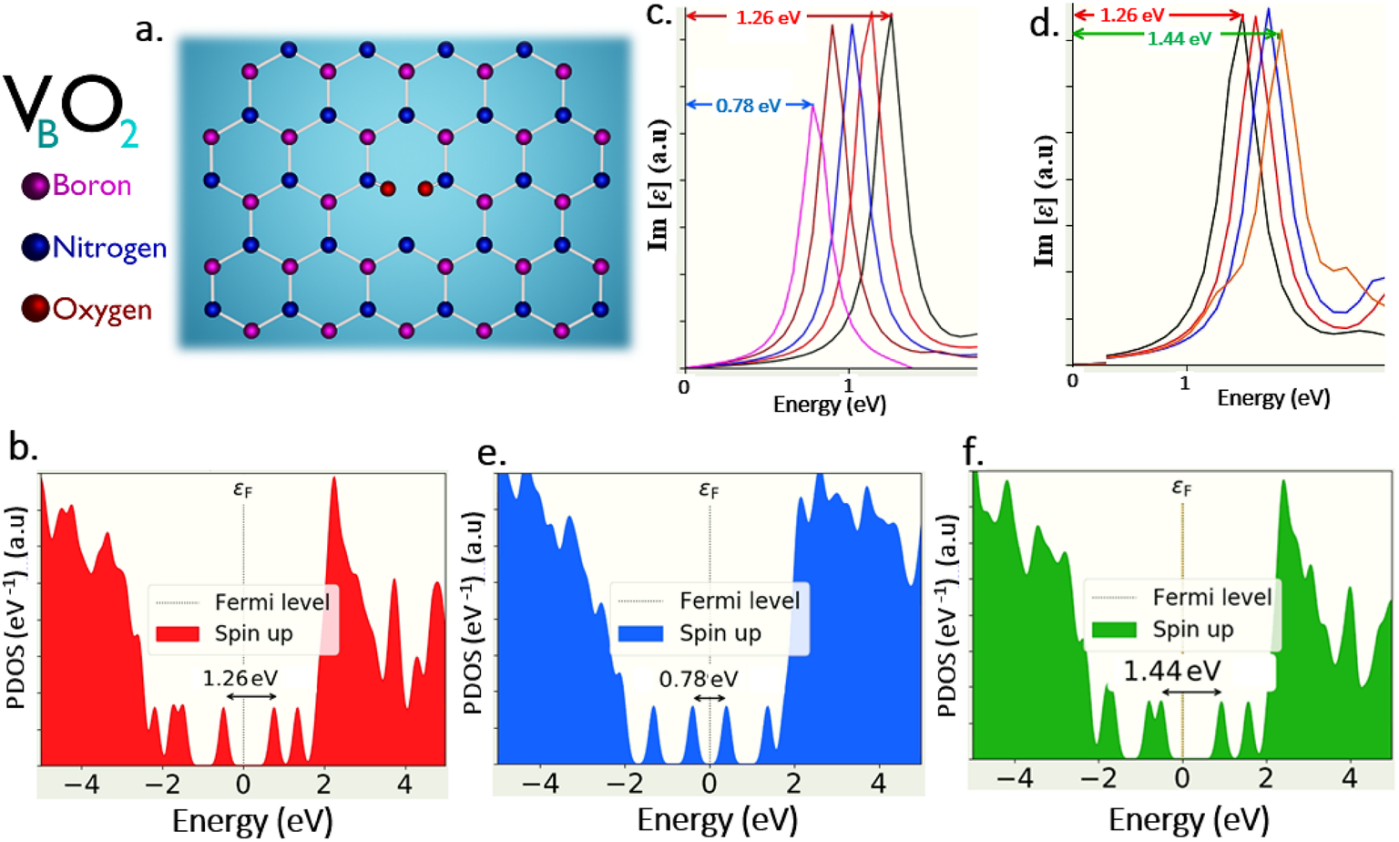
DFT computed V_B_O_2_ defect, their tunable quantum emission towards lower and higher energy regions and corresponding PDOS. (**a**) Schematic illustration of V_B_O_2_ (boron mono vacancy with two oxygen atoms) whose ZPL energy is observed at 1.26 eV. (**b**) PDOS of V_B_O_2_ defect under no external strain, whose possible electronic transition energy difference between inter energy states is consistent with ZPL energy (1.26 eV), which ensures the quantum emission. (**c**,**d**) Quantum emission tunability towards lower and higher energy regions for applied tensile and compressive effects of one-sided longitudinal strain inducements. Red colour arrow indicates the quantum emission peak (ZPL) at 1.26 eV under no strain condition. Blue colour arrow indicates tuned quantum emission peak (tuned ZPL) up to 0.78 eV (towards lower energy region) for applied tensile effect of one-sided longitudinal strain inducement. Green colour arrow indicates tuned quantum emission peak (tuned ZPL) up to 1.44 eV (towards higher energy region) for applied compressive effect of one-sided longitudinal strain inducement. The colours were reflected in PDOS information also. Optical spectrum graphs were obtained by assigning y-axis to imaginary component of dielectric constant [**ε**] and x-axis to energy (eV). (**e**,**f**) Corresponding PDOS of tensile and compressive effects of one-sided longitudinal strained V_B_O_2_ defect, whose energy differences consistent with tuned quantum energies, which ensures the efficient tunability of quantum emission and bandgap modulation.

The schematic of V_B_O_2_ (boron vacancy with oxygen atoms passivated) defect, as shown in Fig. 9a, whose DFT simulation obtained ZPL emission energy and corresponding PDOS are listed in Table 2.

The graphically represented PDOS of V_B_O_2_ defect under no external strain is shown in Fig. 9b, in which the electron occupied ground state lies below (left) to the fermi level line and electron un-occupied excited state lies above (right) to the fermi level line.

These intermediate energy states are formed due to V_B_O_2_ defect and the disclose of Lorentzian shaped ZPL peak at its respective energy (1.26 eV), whose value consistent with energy difference of intermediate states under no external strain inducement (as shown in graphical representation of PDOS), confirms that V_B_O_2_ is intrinsically a potential quantum emitter at near IR region.

Out of all the three different normal strain inducements which are considered for simulation as discussed in methodology section, V_B_O_2_ exhibits giant tunability towards lower and higher energy regions for one-sided longitudinal strain inducement. The defect tunability range and responsible strain details are listed in Tables 3 and 4.

**Table 3:** Information related to quantum emission tunability range towards lower energy region and responsible external strain induced.

Point defect	Tunability range observed and corresponding optical spectrum information		Responsible external strain type induced (simulated) for tuning the quantum emission	
	Tunability range observed (eV)	Optical spectrum figure no		
			Biaxial strain	One-sided lateral or longitudinal strain
V_B_O_2_	1.26–0.78	Figure 9c	No linear tuning observed	Tensile (stretching) effect towards one-sided longitudinal strain inducement
C_B_V_N_	1.74–1.3	Figure 11b	–	Compression (shrinking) effect towards one-sided longitudinal strain inducement
	1.7–1.22	Figure 8	Positive biaxial strain	–
N_B_V_N_	2–1.38	Figure 11e	–	Compression (shrinking) effect towards one-sided longitudinal strain inducement
	2–1.74	Figure S1b	Positive biaxial strain	–
Boron dangling bonds	3.18–2.7	Figure 11h	Positive biaxial strain	No linear tuning observed
C_B_C_N_	3.54–2.8	Figure 12b		
V_BN_	3.5–3.24	Figure 12e		
V_B_	4.7–4.4	Figure 10c		
V_N_	3.59–3.3	Figure 12h	Negative biaxial strain

**Table 4:** Information related to quantum emission tunability range towards higher energy region and responsible external strain induced.

Point defect	Tunability range observed and corresponding optical spectrum information		Responsible external strain type induced (simulated) for tuning the quantum emission	
	Tunability range observed (eV)	Optical spectrum figure no		
			Biaxial strain	One-sided lateral or longitudinal strain
V_B_O_2_	1.26–1.44	Figure 9d	No linear tuning observed	Compression (shrinking) effect towards one-sided of longitudinal strain inducement
C_B_V_N_	1.74–2.47	Figure 11c	–	Tensile (stretching) effect towards one-sided lateral strain inducement
	1.7–2.05	Figure 8	Negative biaxial strain	–
N_B_V_N_	2–2.57	Figure 11f	–	Tensile (stretching) effect towards one-sided lateral strain inducement
	2–2.21	Figure S1a	Negative biaxial strain	–
Boron dangling bonds	3.18–3.3	Figure 11i	Negative biaxial strain	No linear tuning observed
C_B_C_N_	3.54–3.65	Figure 12c		
V_BN_	3.5–4.08	Figure 12f		
V_B_	4.7–4.86	Figure 10d		
V_N_	3.59–3.9	Figure 12i	Positive biaxial strain	

V_B_O_2_ defect exhibits greater tunability up to 0.78 eV (mid IR region) for tensile (stretching) effect produced at one-sided longitudinal strain inducement and tunability up to 1.44 eV (near IR region) for compressive (shrinking) effect produced at one-sided longitudinal strain inducement and the complete tunability is shown in Fig. 9c,d. We also investigated the PDOS of strain induced (one-sided longitudinal) 2D hBN layers containing V_B_O_2_ defect and we observed modulating the bandgap of inter energy states. For the tensile effect produced in one-sided longitudinal strain inducement, the energy gap between these intermediate states is found to be reduced (engineered to lower energy value) as shown in Fig. 9e. This reduced bandgap energy due to strain engineering is responsible for red-shifted quantum emission from V_B_O_2_ defect towards mid IR region and this reduced energy gap value also consistent with tuned emission energy (simulated optical emission spectrum). These complete details related to PDOS of strained defects and their maximum tunable emission are listed in Table 5.

**Table 5:** Information related to feasible tunability region, PDOS of strained quantum emitters and % of strain induced.

Point defect	Tunable emission region	Maximum tunability observed towards and corresponding PDOS information					
		Max. tunability towards lower energy region, corresponding PDOS and max % of strain induced			Max. tunability towards higher energy region, corresponding PDOS and max % of strain induced		
		Energy (eV)	PDOS figure no	Strain %	Energy (eV)	PDOS figure no	Strain %
V_B_O_2_	Near-IR region	0.78	Figure 9e	1.38	1.44	Figure 9f	− 0.19
C_B_V_N_	Near-IR–visible region	1.3	Figure S3b	− 1.04	2.47	Figure S3c	1.62
C_B_V_N_ (Biaxial strain)		1.22	Figure S2c	3.45	2.05	Figure S2b	− 3.45
N_B_V_N_	Visible region	1.38	Figure S3e	− 1.15	2.57	Figure S3f.	0.92
N_B_V_N_ (Biaxial strain)		1.74	Figure S2f	2.8	2.21	Figure S2e	− 4
Boron dangling bonds	Visible–UV (UV-A) region	2.7	Figure S3h	2.31	3.3	Figure S3i	− 0.81
C_B_C_N_		2.8	Figure S4b	4.50	3.65	Figure S4c	− 1.15
V_BN_	UV (UV-A) region	3.24	Figure S4e	0.23	4.08	Figure S4f.	− 0.46
V_B_	UV-B–UV-C region	4.4	Figure 10e	1.84	4.86	Figure 10f	− 0.92
V_N_	UV (UV-A) region	3.3	Figure S4h	− 0.28	3.9	Figure S4i	1.15

*S1 and S2 indicates supporting information Figs. 1 and 2, respectively.

Point to be noted is that the positive and negative strain % values in the Table 5 indicates positive and negative biaxial strains (in case of biaxial strain inducements). In case of, one-sided lateral and longitudinal strain inducements the positive strain values create tensile effects and negative strain values creates compression effects.

Similarly, for the compressive effect produced in one-sided longitudinal strain, the energy gap between these intermediate states is found to be increased (engineered to higher energy value) as shown in Fig. 9f and this increased bandgap energy due to strain engineering is responsible for blue-shifted quantum emission from V_B_O_2_ defect engineered and this increased energy difference is also consistent with tuned emission energy as shown in Table 5.

Hence, this consistence of un-strained and one sided longitudinal strained engineered intermediated states energy gap (observed from PDOS representation), along with un-tuned and tuned emission spectrums confirms the quantum emission tunability (Mid IR to near IR) from V_B_O_2_ defect.

#### Quantum emission tunability of V_B_, V_N_ and V_BN_ defects in UV region

Only mono and di-vacancy defects (V_B_, V_N_ and V_BN_), which are consistent with experimental declarations as listed in Table 1, revealed the intrinsic quantum emission at different segments of UV region. Selecting such class of defects helps in efficiently tune the quantum emission towards deep UV region.

**Figure 10:**
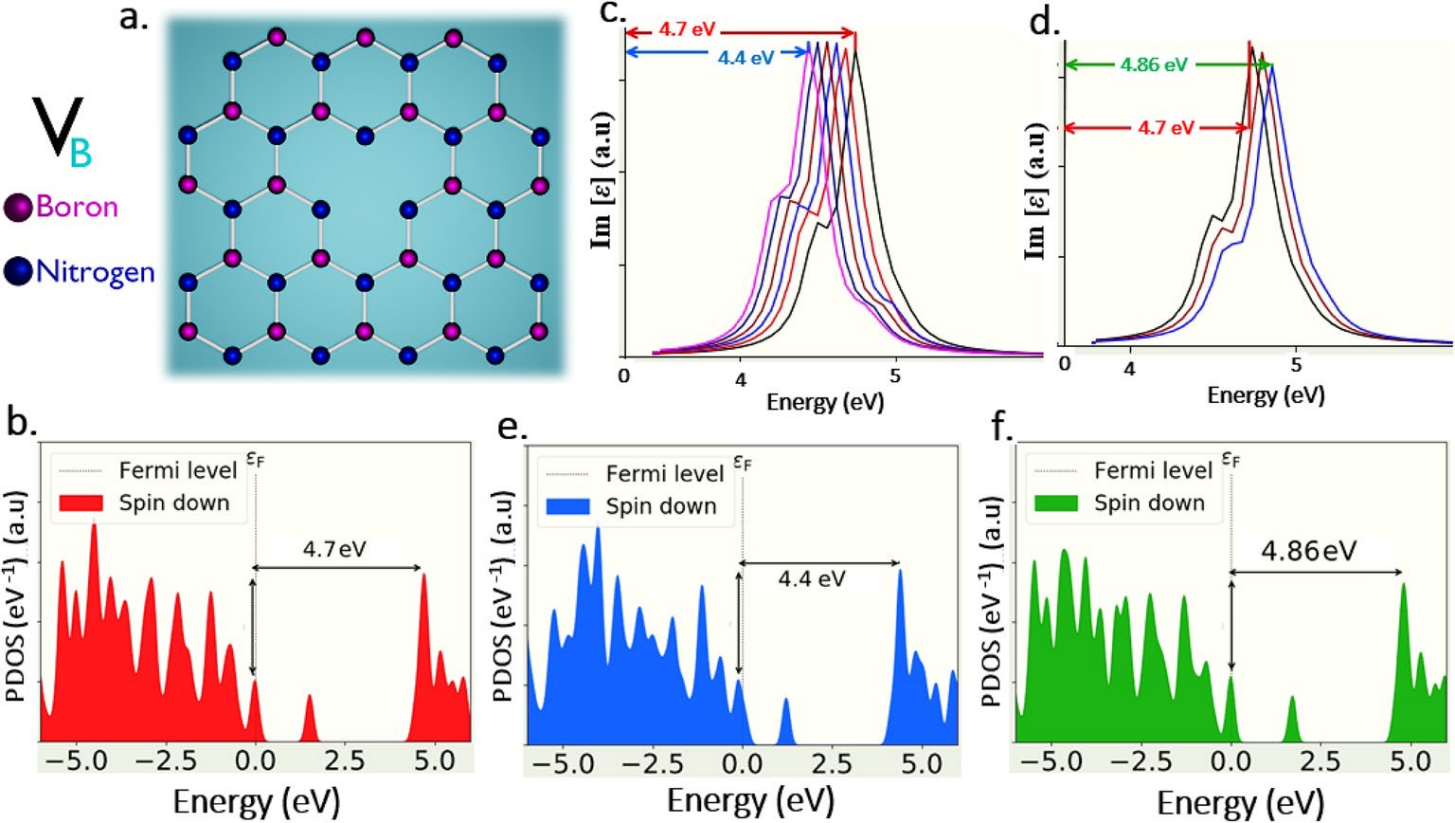
DFT computed V_B_ defect, their tunable quantum emission towards lower and higher energy regions and corresponding PDOS. (**a**) Schematic illustration of V_B_ (boron mono vacancy) whose ZPL energy is observed at 4.7 eV. (**b**) PDOS of V_B_ defect under no external strain, whose possible electronic transition energy difference between inter energy states is consistent with ZPL energy (4.7 eV), which ensures the quantum emission. (**c**,**d**) Quantum emission tunability towards lower and higher energy region for applied positive and negative biaxial strains respectively. Red colour arrow indicates the quantum emission peak (ZPL) at 4.7 eV under no strain condition. Blue colour arrow indicates tuned quantum emission peak (tuned ZPL) up to 4.4 eV (towards lower energy region) for applied positive biaxial strain. Green colour arrow indicates tuned quantum emission peak (tuned ZPL) up to 4.86 eV (towards higher energy region) for applied negative biaxial strain. The colours were reflected in PDOS information also. Optical spectrum graphs were obtained by assigning y-axis to imaginary component of dielectric constant [**ε**] and x-axis to energy (eV). (**e**,**f**) Corresponding PDOS of positive and negative biaxially strained V_B_ defect, whose energy differences consistent with tuned quantum energies, which ensures the efficient tunability of quantum emission and bandgap modulation.

The schematics of V_B_ (boron mono vacancy), V_N_ (nitrogen mono vacancy) and V_BN_ (boron and nitrogen di-vacancy) defects as shown in Figs. 10a, 12g,d respectively, whose DFT simulation obtained ZPL emission energy and corresponding PDOS are listed in Table 2.

The graphically represented PDOS of V_B_, V_N_ and V_BN_ defects under no external strain is shown in Fig. 10b and Fig. S4g,d respectively, disclose the presence of intermediate energy states (in which the electron occupied ground state lies below (left) to the fermi level line and electron un-occupied excited state lies above (right) to the fermi level line), whose energy gaps are consistent with ZPL emission at respective energies (4.7 eV, 3.59 eV and 3.5 eV).

This consistence of PDOS energy gaps of this mono and di-vacancy defects with their Lorentzian shaped ZPL energies confirms their intrinsic potential quantum emission at different shades of UV region.

All these mono and di-vacancy defects exhibit greater and linear tunability towards lower and higher energy regions, only with biaxial strain inducements and these defects tunability range and type of biaxial strains responsible for tuning are listed in Tables 3 and 4.

V_B_ and V_BN_ defects exhibit tunability towards lower energy region up to 4.4 eV and 3.24 eV respectively as shown in Figs. 10c and 12e for positive biaxial strain inducement, in contrast V_N_ defect exhibits tunability towards higher energy region up to 3.9 eV, as shown in Fig. 12i for positive biaxial strain. Vice versa V_B_ and V_BN_ defects exhibits tunability towards higher energy region up to 4.86 eV and 4.08 eV respectively as shown in Figs. 10d and 12f for negative biaxial strain inducement, whereas V_N_ defect exhibits tunability towards lower energy region up to 3.3 eV as shown in Fig. 12e, for the same negative biaxial strain.

We also investigated the PDOS of strain induced (biaxial strain) 2D hBN layers containing V_B_, V_N_ and V_BN_ defects. For the defects V_B_ and V_BN_, energy gap between inter energy states found to be reduced (engineered to lower energy value) as shown in Fig. 10e and Fig. S4e, respectively, for positive biaxial strain inducement, which is responsible for red-shifted quantum emission. Whereas for V_N_ defect energy gap found to be increased (engineered to higher energy value) as shown in Fig. S4i, for the same positive biaxial strain, due to which quantum emission is blue-shifted.

As in contrast, energy gap between inter energy states found to be increased (engineered to higher energy value) for V_B_ and V_BN_ as shown in Fig. 10f and Fig. S4f respectively, for negative biaxial strain, which is the reason for blue-shifted quantum emission. Whereas for V_N_ defect energy gap found to be decreased (engineered to lower energy value) as shown in Fig. S4h, for the same negative biaxial strain, which leads to quantum emission red-shifted.

Thus, the modulated energy gap values for V_B_, V_N_ and V_BN_ defects also consistent with their tuned emission energies (simulated optical emission spectrum) and these complete details related to PDOS of strained defects and their maximum tunable emissions are listed in Table 5.

Hence, the un-strained (no strain applied) and biaxially strained engineered intermediated states energy gap (observed from PDOS representation) of layered hBN containing V_B_, V_N_ and V_BN_ defects were consistent with un-tuned and tuned emission spectrums.

This consistency confirms the tunable quantum emission in UV-A region from V_N_ and V_BN_ defects and tunable quantum emission from UV-B to UV-C region from V_B_ defect.

#### Quantum emission tunability of N_B_V_N_ defect in visible region

Out of all the point defects (whose DFT approximations are comparable with experimental inspections as listed in Table 2), the only luminescent point defect which manifests its complete tunability in visible region is N_B_V_N_ defect (which is mostly referred luminescent point defect to date).

**Figure 11:**
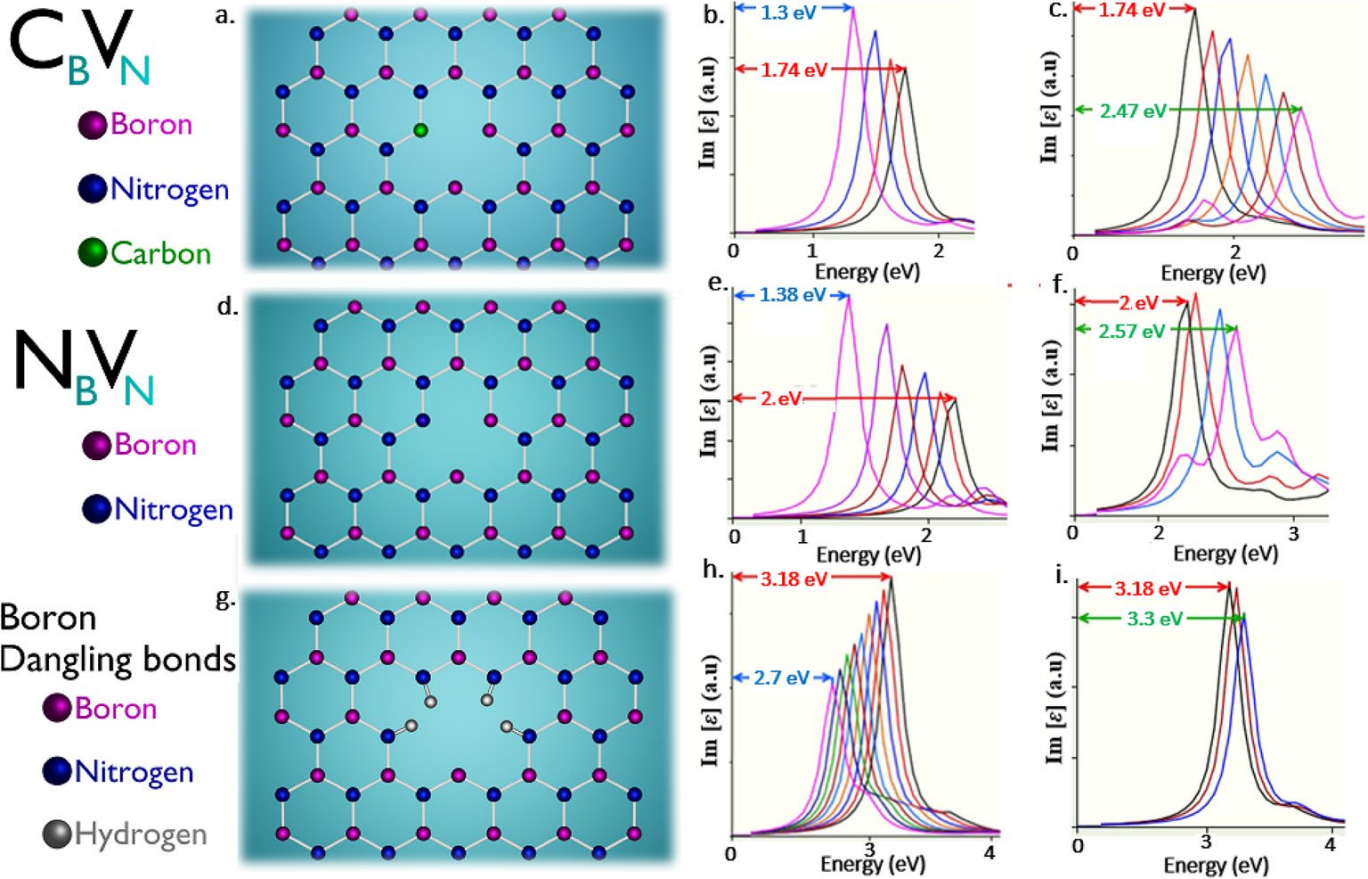
Schematic illustration of DFT computed C_B_V_N_, N_B_V_N_, boron dangling bonds and their corresponding tunable quantum emission towards lower and higher energy regions. (**a**,**d**,**g**) Schematic illustration of C_B_V_N_ (nitrogen mono vacancy with carbon interstitial), N_B_V_N_ (nitrogen mono vacancy with self-interstitial) and boron dangling bonds defects whose ZPL energies are observed at 1.74 eV, 2 eV and 3.18 eV respectively and corresponding tunable quantum emission towards lower and higher energy region for applied one-sided lateral, longitudinal and biaxial strains as described in Tables 3 and 4. (**b**,**e**,**h**) Tunability of defects towards lower energy region. (**c**,**f**,**i**) Tunability of defects towards higher energy region. All red colour arrows indicate ZPL energy under no strain. All blue colour and green colour arrows indicate tuned ZPL energies towards lower energy regions and higher energy regions respectively. The corresponding PDOS information of unstrained and strained quantum emitters were provided in Figure S3 (supporting information).

The schematics of N_B_V_N_ defect (nitrogen mono vacancy with self-interstitial (boron replaced by nitrogen)) as shown in Fig. 11d, DFT simulation obtained ZPL emission energy and corresponding PDOS are listed in Table 2.

The graphically represented PDOS of N_B_V_N_ defect under no external strain is shown in Figs. S2d and S3d, which exhibits the presence of electron occupied and un-occupied intermediate energy states, which are separated by a fermi level line in between. The energy difference between this ground and excited states is consistent with ZPL emission at respective energy (~ 2 eV) and this consistency of PDOS energy gaps with their Lorentzian shaped ZPL energies confirms the clear intrinsic quantum emission in visible region from N_B_V_N_ defect.

The N_B_V_N_ defect responds linearly for all the three different kinds of strains (biaxial, one-sided lateral and one-sided longitudinal strains as discussed in methodology section above). Thus, the defect tunability range for different type of strain inducements and other details are listed in Tables 3 and 4.

The N_B_V_N_ defect exhibits giant tunability towards lower energy region up to 1.74 eV for positive biaxial and 1.38 eV for the compressive effect produced in one-sided longitudinal strain inducements respectively as shown in Fig. S1b and Fig. 11e, respectively.

Besides, the N_B_V_N_ defect exhibits giant tunability towards higher energy region up to 2.21 eV for negative biaxial strain and 2.57 eV for tensile effect produced in on-sided lateral strain inducements respectively as shown in Fig. S1a and Fig. 11f, respectively.

We also examined the PDOS of all three types of strains induced (biaxial, one-sided lateral and one-sided longitudinal) in 2D hBN layers containing N_B_V_N_ defect and we observed giant modulation (strain engineering) of the bandgap of inter energy states. For the positive biaxial strain and for the compressive effect produced in one-sided longitudinal strain inducement, the energy gap between these intermediate states is found to be reduced (engineered to lower energy value) as shown in Figs. S2f and S3e respectively, which is responsible for red-shifted quantum emission from N_B_V_N_ defect.

Contrastingly, for the negative biaxial strain and for the tensile effect produced in one-sided lateral strain inducements, the energy gap between these intermediate states is found to be increased (engineered to higher energy value) as shown in Figs. S2e and S3f respectively, due to which quantum emission from N_B_V_N_ defect is blue-shifted.

Thus, the modulated energy gap values of PDOS for N_B_V_N_ defect (due to biaxial, one-sided lateral and one-sided longitudinal strains) also consistent with their tuned emission energies (simulated optical emission spectrums). These complete details related to PDOS of strained defects and their maximum tunable emissions are listed in Table 5.

Hence, this consistence of un-strained and three different types of strain induced engineered intermediated states energy gaps (observed from PDOS representation), along with un-tuned and tuned emission spectrums confirms the tuning of quantum emission (visible region) from N_B_V_N_ defect

#### Inter-optical band quantum emission tunability

Through-out the discussion of quantum emission tunability till now, all the five different luminescent point defects exhibited their complete possible tunability through a single region (i.e., IR region-V_B_O_2_, UV region—V_B_, V_N_ and V_BN_ and visible region—N_B_V_N_) of electromagnetic spectrum. Moreover, we also found that these three (C_B_V_N_, C_B_C_N_ and boron dangling bonds) point defects can be able to tune the quantum emission, which can cover from near-IR to visible regions and visible to UV (UV-A) regions, respectively.

#### Quantum emission tunability of C_B_V_N_ defect from near-IR to visible region

Among all the luminescent point defects reported to date, (whose DFT calculations are consistent with experimental observations as listed in Table 2), only C_B_V_N_ defect found to exhibit quantum emission tunability from near-IR to visible region.

The schematics of C_B_V_N_ defect (nitrogen mono vacancy with carbon interstitial) as shown in Fig. 11a, DFT simulation obtained ZPL emission energy and corresponding PDOS are listed in Table 2.

The graphically represented PDOS of C_B_V_N_ defect under no external strain is shown in Figs. S2a and S3a, which exhibits the presence of intermediate energy states (electron occupied ground state and un-occupied excited state, separated by a fermi level line in between). The energy difference between these intermediate energy states is consistent with Lorentzian shaped ZPL emission at respective energy (~ 1.7 eV) and this consistency confirms the quantum emission at the frontier of near-IR–visible region, which helps to cover wide tunability towards both near-IR and visible regions.

As similar to N_B_V_N_ defect, C_B_V_N_ defect also responds linearly for all the three different kinds of strains (biaxial, one-sided lateral and one-sided longitudinal strains as discussed in methodology section above). Thus, the defect tunability range for different type of strain inducements and other details are listed in Tables 3 and 4.

C_B_V_N_ defect also exhibits giant tunability towards lower energy region up to 1.22 eV and 1.3 eV respectively as shown in Figs. 8 and 11b, for positive biaxial and for the compressive effect produced in one-sided longitudinal strain inducements and giant tunability towards higher energy region up to 2.05 eV and 2.47 eV respectively as shown in Figs. 8 and 11c, for negative biaxial strain and tensile effect produced in on-sided lateral strain inducements.

We also examined the PDOS of all three types strains induced (biaxial, one-sided lateral and one-sided longitudinal) 2D hBN layers containing C_B_V_N_ defect and we observed greater modulation (strain engineering) of the bandgap of inter energy states. For the positive biaxial strain and for the compressive effect produced in one-sided longitudinal strain inducement, the energy gap between these intermediate states is found to be reduced (engineered to lower energy value) as shown in Figs. S2c and S3b which is responsible for red-shifted quantum emission from C_B_V_N_ defect.

On the other hand, for the negative biaxial strain and for the tensile effect produced in one-sided lateral strain inducements, the energy gap between these intermediate states is found to be increased (engineered to higher energy value) as shown in Figs. S2b and S3c, due to which quantum emission from C_B_V_N_ defect is blue-shifted.

Thus, the modulated energy gap values of PDOS for C_B_V_N_ defect (due to biaxial, one-sided lateral and one-sided longitudinal strains) also consistent with their tuned emission energies (simulated optical emission spectrums) and these complete details related to PDOS of strained defects and their maximum tunable emissions are listed in Table 5.

Hence, this consistence of un-strained and three different types of strain inducing engineered intermediated states energy gaps (observed from PDOS representation), along with un-tuned and tuned emission spectrums confirms the tuning of quantum emission (from near-IR to visible region) from C_B_V_N_ defect.

#### Quantum emission tunability of C_B_C_N_ and boron dangling bonds defects from visible to UV (UV-A) region:

From the herd of luminescent point defects till date, (whose DFT approximations and experimental inspections are consistent with each other as listed in Table 2), only C_B_C_N_ defect and boron dangling bonds found to exhibit tunable quantum emission from visible to UV (UV-A) region.

**Figure 12:**
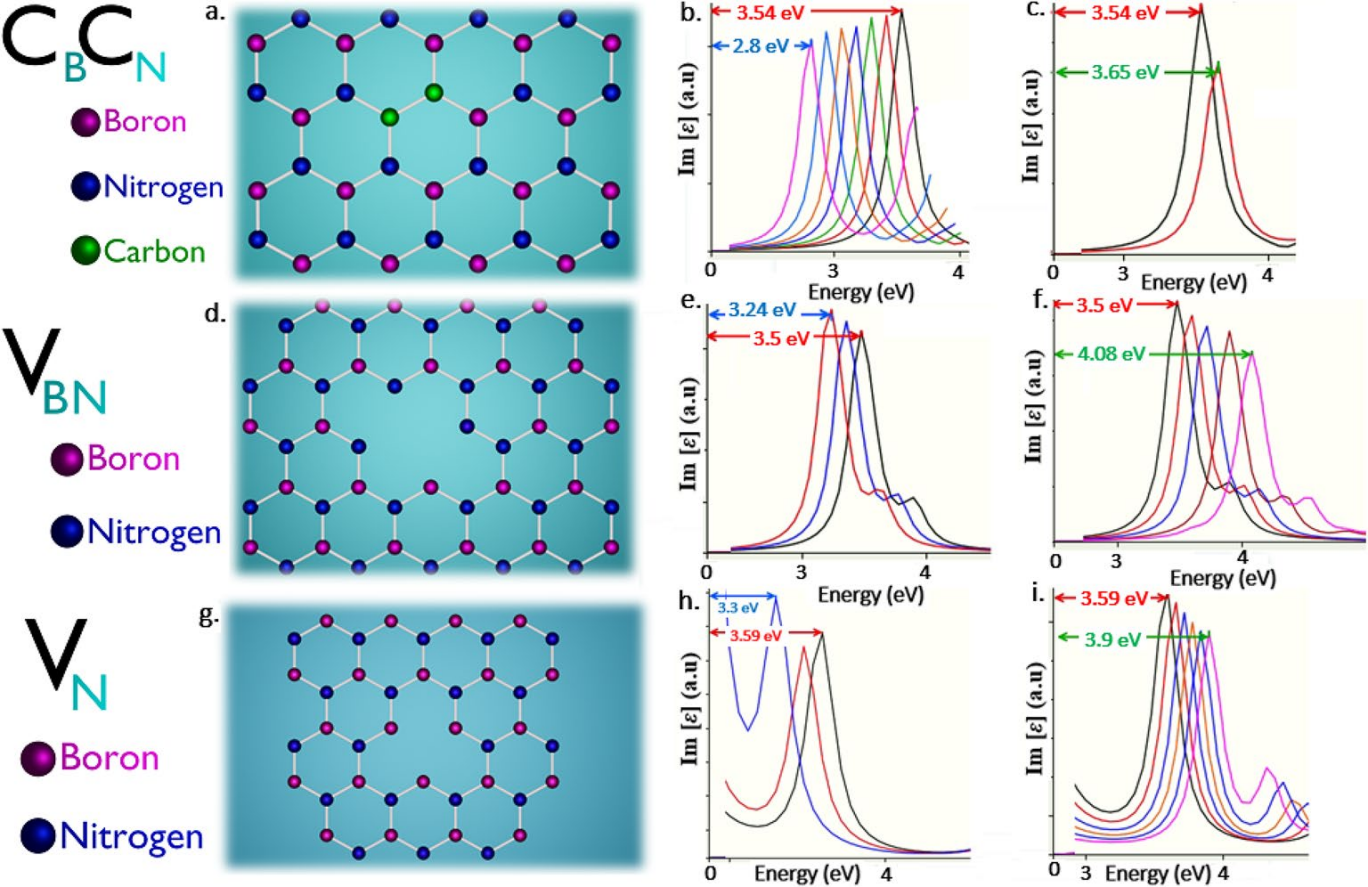
Schematic illustration of DFT computed C_B_C_N_, V_BN_ and V_N_ defects and their corresponding tunable quantum emission towards lower and higher energy regions. (**a**,**d**,**g**) Schematic illustration of C_B_C_N_ (carbon dimer), V_BN_ (boron and nitrogen di-vacancy) and V_N_ (nitrogen mono vacancy) defects whose ZPL energies are observed at 3.54 eV, 3.5 eV and 3.59 eV respectively and corresponding tunable quantum emission towards lower and higher energy region for biaxial strains as described in Tables 3 and 4. (**b**,**e**,**h**) Tunability of defects towards lower energy region. (**c**,**f**,**i**) Tunability of defects towards higher energy region. All red colour arrows indicate ZPL energy under no strain. All blue colour and green colour arrows indicate tuned ZPL energies towards lower energy regions and higher energy regions respectively. The corresponding PDOS information of unstrained and strained quantum emitters were provided in Figure S4 (supporting information).

The schematics of C_B_C_N_ (carbon dimers) and boron dangling bonds defects as shown in Figs. 12a and 11g respectively, DFT simulation obtained ZPL emission energy and corresponding PDOS are listed in Table 2.

The graphically represented PDOS of C_B_C_N_ and boron dangling bonds defects under no external strain is shown in Figs. S4a and S3g, which reveal the presence of intermediate energy states (in which the electron occupied ground state lies below (left) to the fermi level line and electron un-occupied excited state lies above (right) to the fermi level line). The energy difference between these intermediate energy states is consistent with Lorentzian shaped ZPL emission at respective energies (3.54 eV and 3.18 eV) and this consistency confirms the quantum emission at around the borderline of visible–UV region, which helps to cover wide tunability towards both visible and UV (UV-A) regions.

This C_B_C_N_ and boron dangling bonds defects exhibits greater and linear tunability towards lower and higher energy regions, only for biaxial strain inducements and these defects tunability range and type of biaxial strains responsible for tuning are listed in Tables 3 and 4.

The C_B_C_N_ and boron dangling bonds defects exhibits tunability towards lower energy region up to 2.8 eV and 2.7 eV respectively for positive biaxial strain inducement as shown in Figs. 12b and 11h respectively and vice versa the defects exhibit tunability towards higher energy region up to 3.65 eV and 3.3 eV respectively as shown in Figs. 12c and 11i for negative biaxial strain inducement.

We also investigated the PDOS of strain induced (biaxial strain) 2D hBN layers containing C_B_C_N_ and boron dangling bonds defects, whose energy gaps between inter energy states found to be reduced (engineered to lower energy value) as shown in Figs. S4b and S3h respectively, for positive biaxial strain inducement, which is responsible for red-shifted quantum emission. As in contrast, energy gaps between inter energy states found to be increased (engineered to higher energy value) as shown in Figs. S4c and S3i respectively, for negative biaxial strain, which is the reason for quantum emission blue-shifted.

Thus, the modulated energy gap values of PDOS for C_B_C_N_ and boron dangling bonds defects also consistent with their tuned emission energies (simulated optical emission spectrum). These complete details related to PDOS of strained defects and their maximum tunable emissions are listed in Table 5.

Hence, this consistence of un-strained and biaxially strained engineered intermediated states energy gap (observed from PDOS representation), along with un-tuned and tuned emission spectrums confirms the tunable quantum emission (from visible to UV (UV-A) region) from C_B_C_N_ and boron dangling bonds defects.

### Physical interpretation of tunability of quantum emitters for different strain inducements

According to the simulated data obtained, only the V_B_O_2_ defect is found to be potential candidate to tune the quantum emission at telecom wavelength range (C-band) around 1589.5 nm (0.78 eV) as shown in Fig. 9, due to external strain inducement, which is an essential requirement of QKD in IR region and on the other hand, boron mono vacancy (V_B_ defect), exhibit the emission tunability deep into solar bind (UV-C) region around 255 nm (4.86 eV) as shown in Fig. 10, which strongly supports and enhances the efficient implementation of quantum communication in UV region.

Rest of the defects exhibit their tunable emission at visible-IR region (C_B_V_N_ defect), UV–visible region (boron dangling bonds and C_B_C_N_ defects) and around the parts of UV-A region (V_N_ and V_BN_ defects) as shown in Figs. 8, 11, and 12.

Only N_B_V_N_ defect exhibits the tunability range through the core of visible region as shown in Fig. S1 and Fig. 11e,f and such intermittent emissions may promote the enhancement of quantum photonic devices. The short summary table extracted from analysis of results and discussion section, which provides a quick information and comparison about luminescent point defects and the responsible external strain inducement types, for tuning towards lower and higher energy regions is shown in Table 6.

**Table 6:** Summary table of tunability of various point defects for different external strain inducements.

Point defects	Responsible strain for tunability of quantum emission towards lower and higher energy regions		No tuning observed
	Tunability towards lower energy region	Tunability towards higher energy region	
V_B_O_2_	Tensile (stretching) effect towards one-sided longitudinal strain inducement	Compression (shrinking) effect towards one-sided of longitudinal strain inducement	Biaxial strain
V_B_O_2_ defect exhibits tunability towards lower and higher energy regions due to tensile and compressive effects of one-sided longitudinal strain inducements respectively			
C_B_V_N_ N_B_V_N_	Compression (shrinking) effect towards one-sided longitudinal strain inducement	Tensile (stretching) effect towards one-sided lateral strain inducement	––––
	Positive biaxial strain	Negative biaxial strain	
C_B_V_N_ and N_B_V_N_ defects exhibits tunability towards lower energy region due to positive biaxial and compressive effect of one-sided longitudinal strain inducements and tunability towards higher energy region due to negative biaxial and tensile effect of one-sided lateral strain inducements			
Boron dangling bonds	Positive biaxial strain	Negative biaxial strain	One-sided lateral and longitudinal strains
C_B_C_N_			
V_BN_			
V_B_			
For positive biaxial strain, boron dangling bonds to V_B_ defects exhibits tunability towards lower energy region and for negative biaxial strain, boron dangling bonds to V_B_ defects exhibits tunability towards higher energy region			
V_N_	Negative biaxial strain	Positive biaxial strain	One-sided lateral and longitudinal strains
For negative biaxial strain, V_N_ defect exhibits tunability towards lower energy region and for positive biaxial strain, V_N_ defect exhibits tunability towards higher energy region			

From the simulated data, it was observed that wide range of tunability is observed for all the defects (V_B_O_2_, C_B_V_N_, N_B_V_N_, C_B_C_N_, V_BN_ and boron dangling bonds) than compared to mono vacancy defects (V_B_ and V_N_). For all the defects as listed Table 1, more tunability is observed towards lower energy region compared to higher energy region, except V_BN_ and C_B_V_N_ defects which exhibited greater tunability towards higher energy region compared to lower energy region as observed in Tables 3 and 4.

In particular, we observed only nitrogen mono vacancy with self or carbon interstitial (N_B_V_N_ and C_B_V_N_) defects exhibits giant tunability for all the three different strains (biaxial, one-sided lateral and one-sided longitudinal strains). In particular, greater order of tunability is observed for N_B_V_N_ and C_B_V_N_ defects for one-sided lateral and one-sided longitudinal strains than compared to biaxial strains as observed in Refs.^14,21,25^.

The quantum emitters found to hold its ZPL Lorentzian shape (signature of quantum emission) without deforming, throughout the possible tunability range from lower to higher energy region, for all the strains induced.

Different luminescent point defects exhibit different tunability range and different respondence for different strain values, which further confirms that each luminescent point defect has its own unique characteristics such as different fabrication processes and defect structure orientations, different energy gaps between intermediate energy states (created due to point defects), etc.

While tuning the quantum emission towards lower and higher energy regions by inducing various external strains, we observed that, all of the defects exhibit either increase or decrease in intensities of Lorentzian shaped ZPL peaks in the optical emission spectrums. This increase or decrease in intensities while tuning the quantum emission was also observed in earlier experiments and DFT computational works^14,21^.

Conventionally, this ZPL intensity represents the emission rate (photons emitted per second)^39^ and this increase or decrease in intensity will convey the increase or decrease in emission rate, while quantum tuning by external strain inducement.

In elucidation at atomic level, the significant reason behind this shifting and tuning of emission spectrum from luminescent point defects is due to its alter and deformation of crystal structure due to external strain inducements.

Predominantly, we have observed the deformation in atomic bond lengths of luminescent point defects for different strain inducements. In this article, we present some of the point defects whose bond lengths are deformed due to external strain inducements, which are responsible for their tuning their emission spectrum towards lower and higher energy regions.

**Figure 13:**
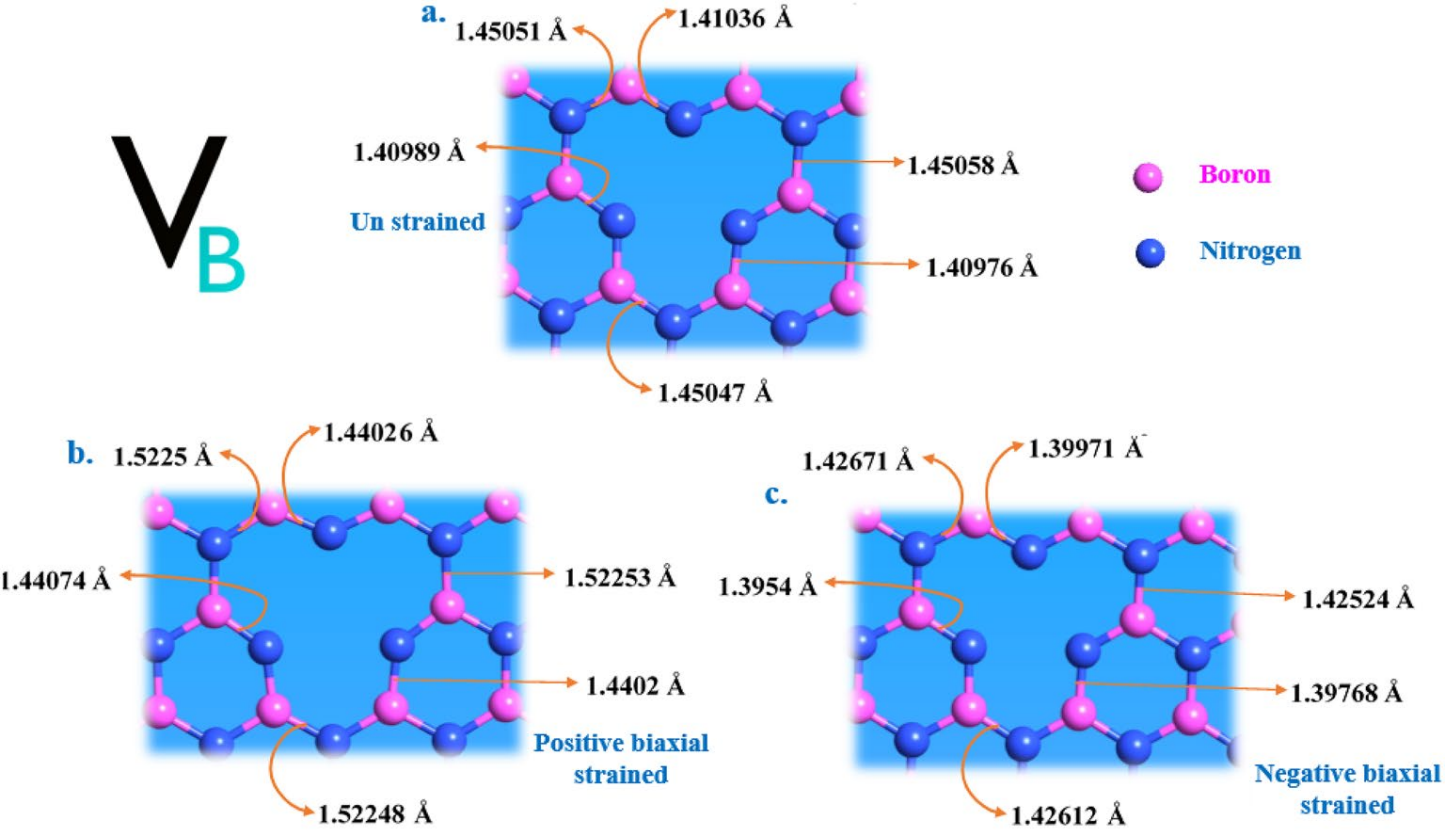
Schematic representation of bond length alters in V_B_ defect due to biaxial strain inducement. (**a**) Optimized V_B_ point defect and their corresponding bond lengths measured under no external inducement condition. (**b**,**c**) V_B_ point defects and their altered bond lengths measured under positive and negative biaxial strain inducements, respectively. The bond lengths found incremented for positive biaxial strain and decremented for negative biaxial strain inducements, compared to bond lengths of point defect under no strain condition as shown in (**a**). The % of positive and negative strain applied to the V_B_ defect was listed in Table 5.

Figure 13 represents the deformation of bond lengths for V_B_ point defect, due to biaxial strain inducements. Figure 13a represents the optimized crystal structure of V_B_ defect and their corresponding bond lengths, under zero strain condition (no external strain induced).

Figure 13b represents the deformation of V_B_ point defect crystal bond lengths due to positive biaxial strain inducements. Figure 13c represents the deformation of V_B_ point defect crystal bond lengths due to negative biaxial strain inducements.

As the change in bond lengths were clearly observed, we can find that for positive biaxial strain inducements the crystal bond lengths were found to increase than compared to bond lengths of point defects (under no strain conditions). This increase in bond lengths can be significant reason for point defects bandgap modulation (decrement in bandgap) and corresponding shift of emission spectrum towards lower energy region.

Similarly, for negative biaxial strain inducements the crystal bond lengths were found to decrease than compared to bond lengths of point defects (under no strain conditions). This decrease in bond lengths can be significant reason for point defects bandgap modulation (increment in bandgap) and corresponding shift of emission spectrum towards higher energy region.

We also examined the crystal bond length deformation of point defects (boron dangling bonds, N_B_V_N_ and C_B_V_N_) for positive and negative biaxial strain inducements and the variation in bond length schematics was presented in Figs. S5, S6 and S7, respectively.

For the point defects (boron dangling bonds, N_B_V_N_ and C_B_V_N_), we have observed the increment in bond lengths for positive biaxial strain and decrement in bond lengths for negative biaxial strain, same as the behaviour observed for V_B_ point defect as shown in Fig. 13.

As per our surveillance, the symmetrical relation of red-shifted quantum emission for increase in all bond lengths of point defects (boron dangling bonds, VB, N_B_V_N_ and C_B_V_N_) due to positive biaxial strain and blue-shifted quantum emission for decrease in all bond lengths of point defects due to negative biaxial strain inducements was observed.

But this symmetry was broken in case of one-sided lateral and longitudinal strain inducements. This is due to the negative Poisson’s ratio effect involved along one-sided lateral and longitudinal strain inducements as explained at Figs. 3 and 7.

**Figure 14:**
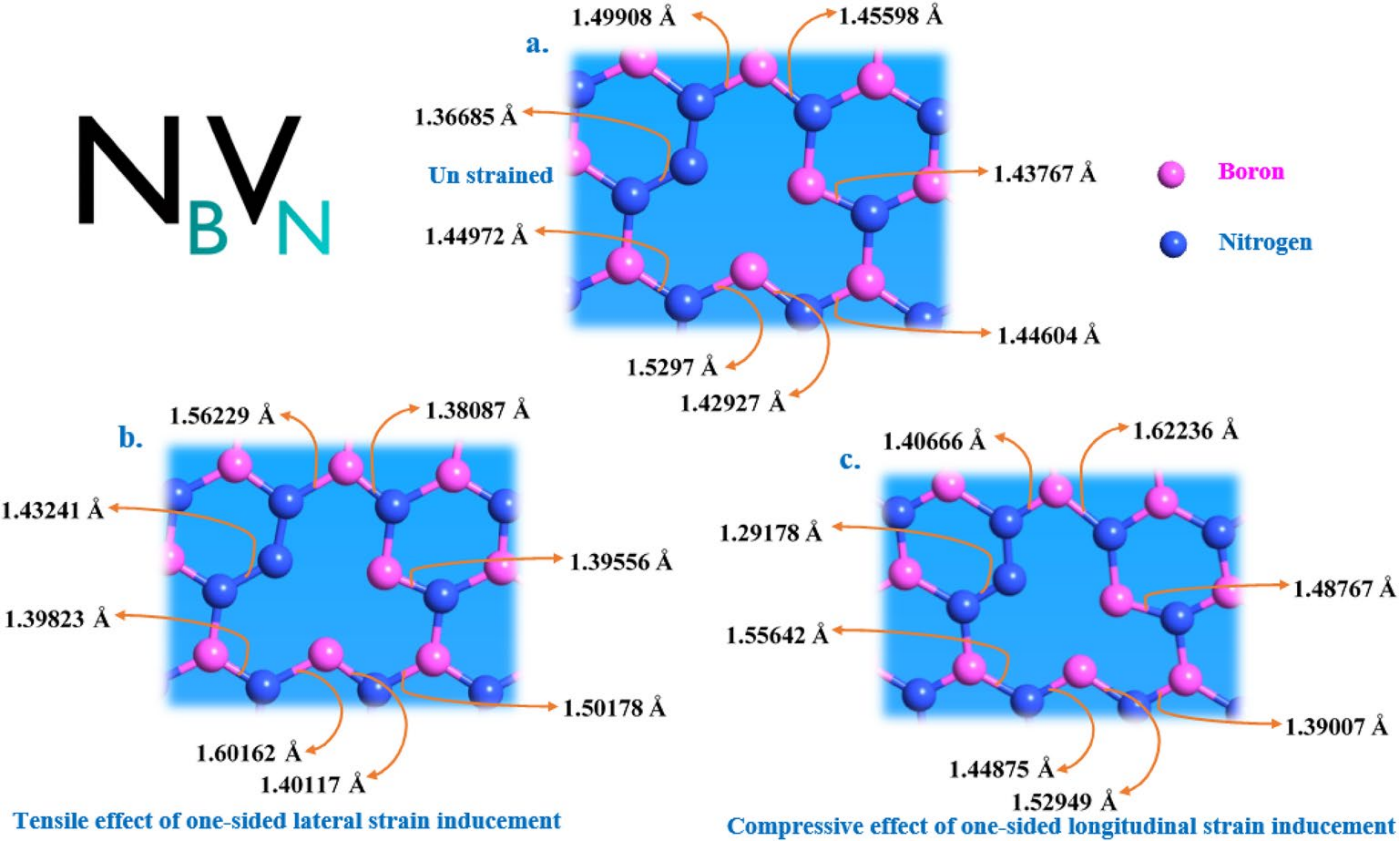
Schematic representation of bond length alters in N_B_V_N_ defect due to one-sided lateral and longitudinal strain inducement. (**a**) Optimized N_B_V_N_ point defect and their corresponding bond lengths measured under no external inducement condition. (**b**,**c**) N_B_V_N_ point defect and their altered bond lengths measured under tensile effect of one-sided lateral strain and compressive effect of one-sided longitudinal strain inducements. Some of the bond lengths tends to increase and some of the bond lengths tends to decrease in both one-sided lateral and longitudinal strain inducements. This is due to the involvement of Poisson’s ratio effect along with one-sided lateral and longitudinal strain inducements. The % of positive and negative strain applied to the N_B_V_N_ defect was listed in Table 5.

We have observed the bond length deformation for the tensile and compression effects of one-sided lateral and longitudinal strain inducements for the point defects N_B_V_N_ and C_B_V_N_ as shown in Fig. 14 and Fig. S8, respectively.

For the tensile effect of one-sided lateral strain inducement for N_B_V_N_ and C_B_V_N_ defects, some of the bond lengths tends to increase and some of the bond lengths tends to decrease as shown in Fig. 14b and Fig. S8b, respectively. This both increase and decrease in bond lengths are due to tensile effect of one-sided lateral strain and corresponding compressive Poisson’s ratio deformation.

Similarly, for the compressive effect of one-sided longitudinal strain inducement for N_B_V_N_ and C_B_V_N_ defects, some of the bond lengths tends to increase and some of the bond lengths tends to decrease as shown in Fig. 14c and Fig. S8c, respectively. This both increase and decrease in bond lengths are due to compressive effect of one-sided longitudinal strain and corresponding tensile Poisson’s ratio deformation.

### Spin preserved transitions of luminescent point defects

As discussed in above sessions, these luminescent point defects create an intermediate energy states in between wide bandgap (between valence and conduction bands) of 2D hBN as shown in Ref.^39^. When this occupied ground state electron is excited with enough energy, it transmits to un-occupied excited state. During this transition, the single electron found to preserve its own individual spin, i.e., the electron transmits to the excited state, which possess the same spin type of electron.

This spin type can be either spin-up ↑ or spin-down ↓ and all luminescent point defects (as listed in Table 1), found to preserve different spin polarized transitions and information related to type of spin transition does point defect preserve can be obtained from PDOS execution of luminescent point defects. The complete data related to the type of spin transition, point defects has preserved during our DFT simulations were listed in Table ST1 (tabulated in supportive information).

### Excited states discussion from other higher-level approximations

Our DFT computations can provide valuable insights related to luminescent point defects quantum emission energies, tunable emission ranges due to various external strain inducements and corresponding density of states occupation in intermediate energy levels. But, constrained to address the important aspects of luminescent point defects such as excited states structures^45^ of point defects, spin- orbit and their hyperfine couplings.

The state-of-the-art, GW approximations with Bethe–Salpeter equation (BSE) and other recent methods could accurately characterize the properties of luminescent point defects such as excited states structures, spin–orbit and hyperfine couplings, which is beyond the ability of DFT studies^31–35^. Yet, these first principles calculations of luminescent point defects, using GW approximations with BSE calculations are a recent advance, these demand high computational resources and time. Hence in this work, we address some of the earlier GW calculations of point defects, whose approximations are almost consistent with our work.

Mono and di-vacancy defects whose emissions are found to be around 4 eV, revealed the presence of electron fully occupied, half-filled and un-occupied energy states in excited state’s structure calculations using GW approximations as shown in Ref.^46^, whose transitions resemble single photon emission possibilities. As per our DFT simulations, V_B_ defect exhibits greater tunability towards deep solar blind (UV-C) region and their corresponding hyperfine coupling of degenerate states were also computed using GW-BSE calculations also estimated recently as in Refs.^47,48^.

The accuracy of defect levels was examined by calibrating the individual levels against ab-initio CCSD(T), EOMCCSD, CASPT2, and MRCI calculations^49^ and in such process spin–orbit and hyperfine coupling of C_B_V_N_ defect were also examined as in Ref.^49^. Contemporary findings from GW approximations were that, as similar to other 2D materials and their corresponding defects, the optical transitions associated to the defects in hBN are also dominated by excitonic effects^50^. Among the pool of defects, only N_B_V_N_ defect exhibits highest likelihood in correlating their atomic structure with photophysical characteristics as per GW-BSE approximations^50^. These deep calculations revealed, the strong dependence of its radiative properties on the small perturbations of the atomic structure of N_B_V_N_ defect.

### Spin un-polarized versus polarized quantum emission tunability of point-defects

**Figure 15:**
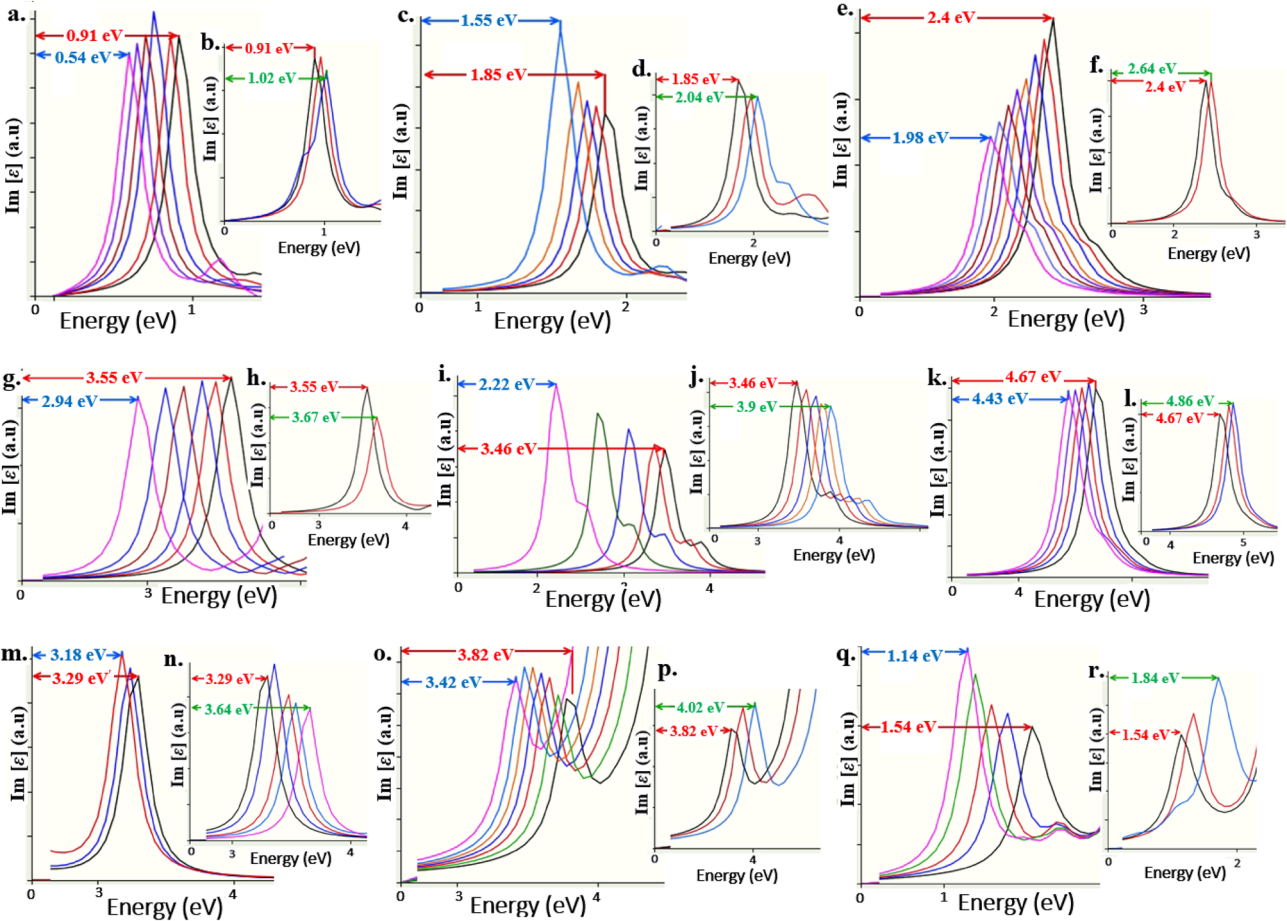
DFT computed, spin un-polarized quantum emission tunability of luminescent point defects to external strain inducement. All the emission spectrums towards left panel are quantum emission tunability towards lower energy region and emission spectrums towards right panel are tunability towards higher energy region. (**a**,**b**) Tunability of V_B_O_2_ defect having ZPL at 0.91 eV. (**c**,d) Tunability of N_B_V_N_ defect having ZPL at 1.85 eV. (**e**,**f**) Tunability of boron dangling bonds having ZPL at 2.4 eV. (**g**,**h**) Tunability of C_B_C_N_ defect having ZPL at 3.55 eV. (**i**,**j**) Tunability of V_BN_ defect having ZPL at 3.46 eV. (**k**,**l**) Tunability of V_B_ defect having ZPL at 4.67 eV. (**m**,**n**) Tunability of V_N_ defect having ZPL at 3.29 eV. (**o**,**p**) Tunability of C_B_ defect (boron replaced by carbon) having ZPL at 3.82 eV. (**q**,**r**) Tunability of N_AL_V_N_ defect in AlN (aluminum nitride) having ZPL at 1.54 eV. All red colour arrows indicate ZPL energy under no strain. All blue colour and green colour arrows indicate tuned ZPL energies towards lower energy regions and higher energy regions, respectively.

We re-performed the strain inducement simulations by setting all the DFT simulation parameters to same, but assigning the spin to un-polarized conditions and we obtain all most similar tunability of quantum emitters with small variations in emission energies and their tunable quantum emission were shown in Fig. 15.

Earlier literatures reported that boron replaced by carbon (C_B_ defect)^51^, is also an efficient UV quantum emitter. Hence, we also examined the tunability of C_B_ defect through external strain, and as predicted C_B_ defect exhibits greater tunability towards lower energy region compared to its tunability towards higher energy region as shown in Fig. 15o,p.

For C_B_ defect, well isolated ZPL Lorentzian shape (quantum emission signature) was observed for un-polarized spin calculations than compared to spin polarized DFT computations. In order to examine the stretchability property of 2D materials, we also simulated positive and negative biaxial strain inducement simulations (under spin un-polarised conditions) on N_AL_V_N_ defect^52^ formed in 2D AlN (aluminum nitride) material and we obtained the tunability as observed in Fig. 15q,r, which confirm the high stretchability property of 2D materials.

## Conclusion

To summarize, we confirm that quantum emitters in layered hBN are the potential candidates to cover the single photon emission from telecom (C-band) wavelength range to solar blind (UV-C) region. The natural hyperbolic and high stretchability properties of 2D hBN, allows to induce controllable external strain to high orders and customize the quantum emission wavelength. By constrained DFT computations, we simulated inducement of biaxial and constrained normal strains (one-sided lateral and one-sided longitudinal strains) to the quantum emitters, which are considered as promising single photon sources. Particularly, V_B_O_2_ and V_B_ defects found to exhibit the quantum emission tunability for the external strain, to the wavelength range of 1589.5 nm and 255 nm respectively, which strengthens the successful implementation of quantum key distribution for longer and short-range distances via optical fibers and free-space channels, respectively. Rest of significant quantum emitters (boron dangling bonds, C_B_V_N_, C_B_C_N_, V_BN_ and V_N_ defects) reveal the quantum emission tunability from UV-A to near-IR region and solitarily N_B_V_N_ defect projects quantum emission tuning through the core of visible region, which enhances the implementation of quantum photonic devices. The corresponding PDOS graphs of unstrained and strained luminescent point defects gives a back support to confirm the quantum emission tunability through external strain inducement. And to the great extent, irrespective of its time-consuming, a more complex GW calculations approximation of all luminescent point defects is necessary to more accurately characterize the properties such as excited state’s structure, spin–orbit and hyperfine couplings etc. Further, experimental validation is needed to practically assess this quantum emission tunability and inspect their single photon purity and the properties as mentioned above. Our outcomes may advise the experimentalists the practical implementation of quantum emitters with customized emission wavelength or hammer out absolute UV-C to telecom (C-band) wavelength emission on a single host material, which enhances to establish the robust QKD based quantum information technologies.

## Supplementary Information

Supplementary Information.

## Data Availability

The datasets used and/or analysed during the current study available from the corresponding author on reasonable request.
