# Supplementary material for: Strain tunable quantum emission from atomic defects in hexagonal boron nitride for telecom-bands
Source: Sci Rep. 2022 Dec 15;12:21673. doi: 10.1038/s41598-022-26061-w (PMC9755526; doi:10.1038/s41598-022-26061-w)
Supplement: Supplementary file 1 — Supplementary Information. [file 41598_2022_26061_MOESM1_ESM.pdf]

Supporting information

Strain Tunable Quantum Emission from Atomic Defects in Hexagonal Boron Nitride for Telecom-bands

Akbar Basha Dhu-al Shaik[1] and Penchalaiah Palla[2]*

[1, 2]Department of Micro and Nanoelectronics, School of Electronics Engineering,

Vellore Institute of Technology, Vellore, Tamil Nadu, 632014, India.

*Corresponding author email: penchalaiah.palla@vit.ac.in (official),

pench.palla@gmail.com (personal)

I. Biaxial tuning of N_BV_N defect:

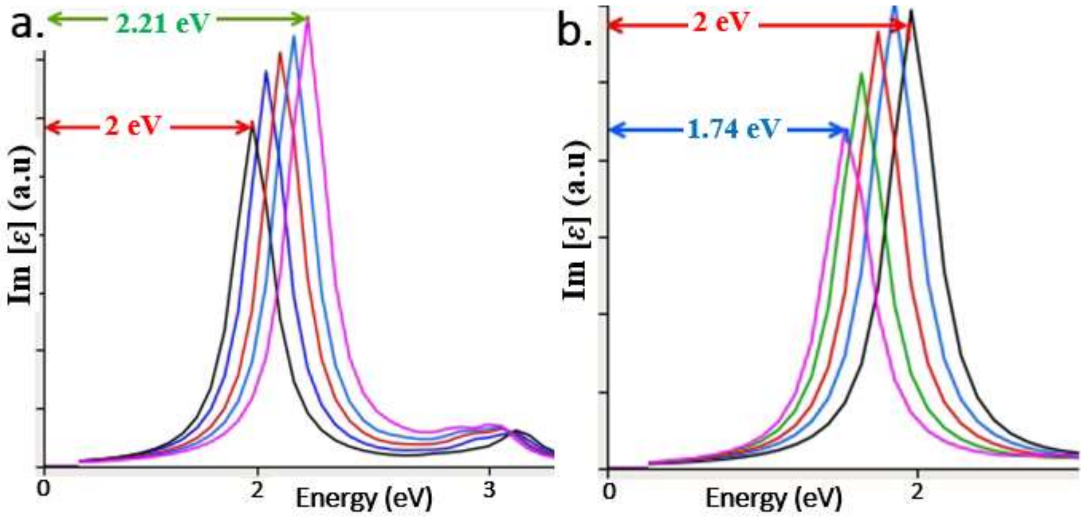

Figure S1: Biaxial tuning of N_BV_N defect. The N_BV_N defect whose ZPL quantum emission is observed around 2 eV (under on external strain condition), which is represented in red colour arrow. (a.) Quantum ZPL emission is blue-shifted for negative biaxial strain inducement, which is represented by green colour arrow. (b.) Quantum ZPL emission is red-shifted for positive biaxial strain inducement, which is represented by blue colour arrow. We induced the strain – 4% to + 2.8% for negative and positive biaxial strain inducement simulations. The DFT computations were performed using plane-wave calculations as explained in methodology section.

`

II. PDOS of un-strained and Biaxially strained C_BV_N and N_BV_N defects:

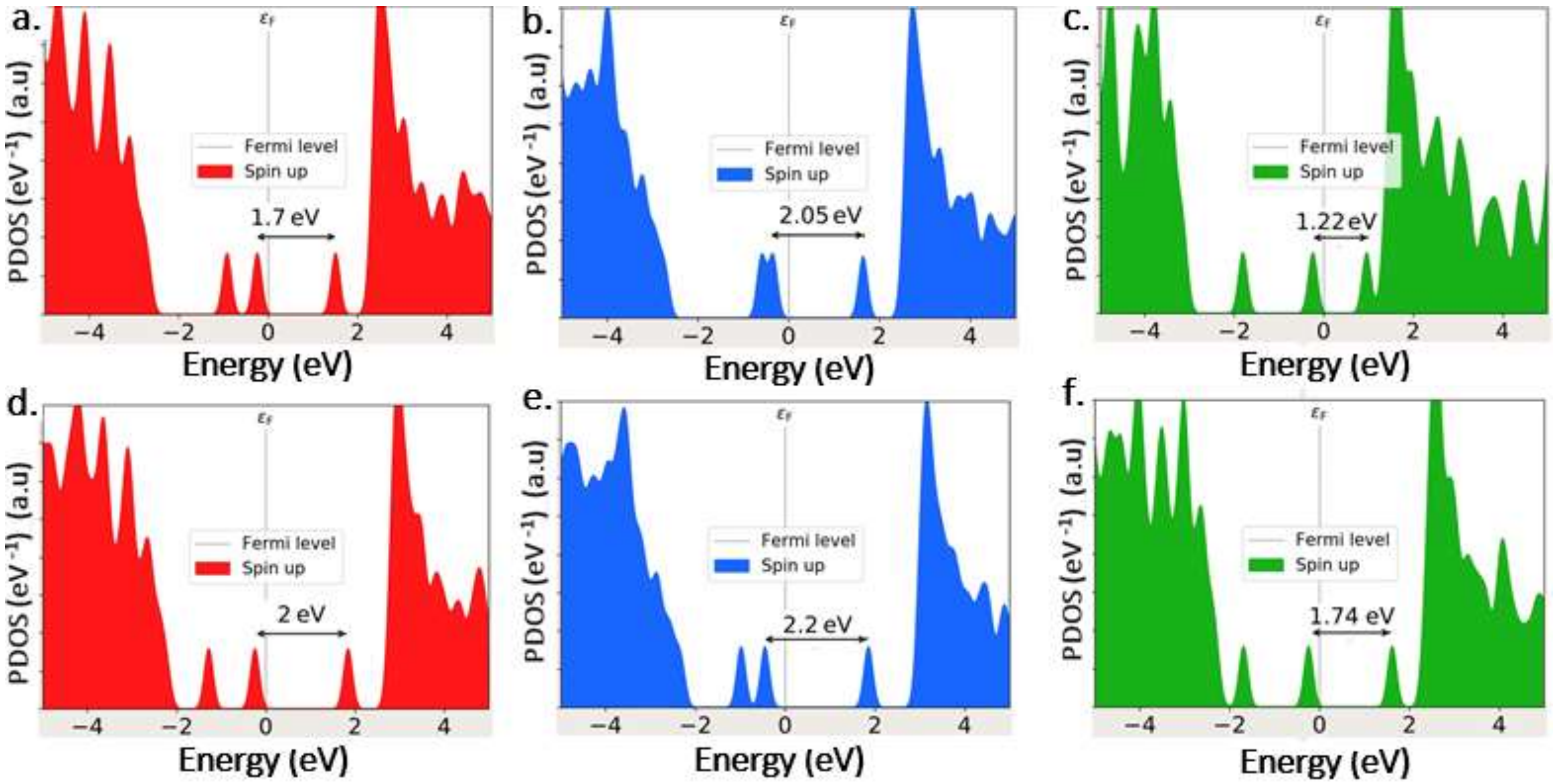

Figure S2: PDOS of C_BV_N, N_BV_N defects under un-strained and biaxially strained conditions. (a, d) Red coloured graphs are PDOS of quantum emitters (C_BV_N and N_BV_N defects) under un-strained conditions, whose electronic transition energy difference between inter energy states is consistent with ZPL energies and ensures the quantum emission. (b, e) Blue coloured graphs are PDOS of negative biaxially strained quantum emitters, whose emission is tuned towards higher energy region and tuned ZPL energies are consistent with inter-state energy differences, which confirms the quantum emission alter. (c, f) Green coloured graphs are PDOS of positive biaxially strained quantum emitters, whose emission is tuned towards lower energy region and tuned ZPL energies are consistent with inter-state energy differences, which confirms tuning of quantum emission. The DFT strain inducement calculations for obtaining this PDOS were performed using plane-wave calculations as explained in methodology section.

`

III. PDOS of un-strained and other strained quantum emitters:

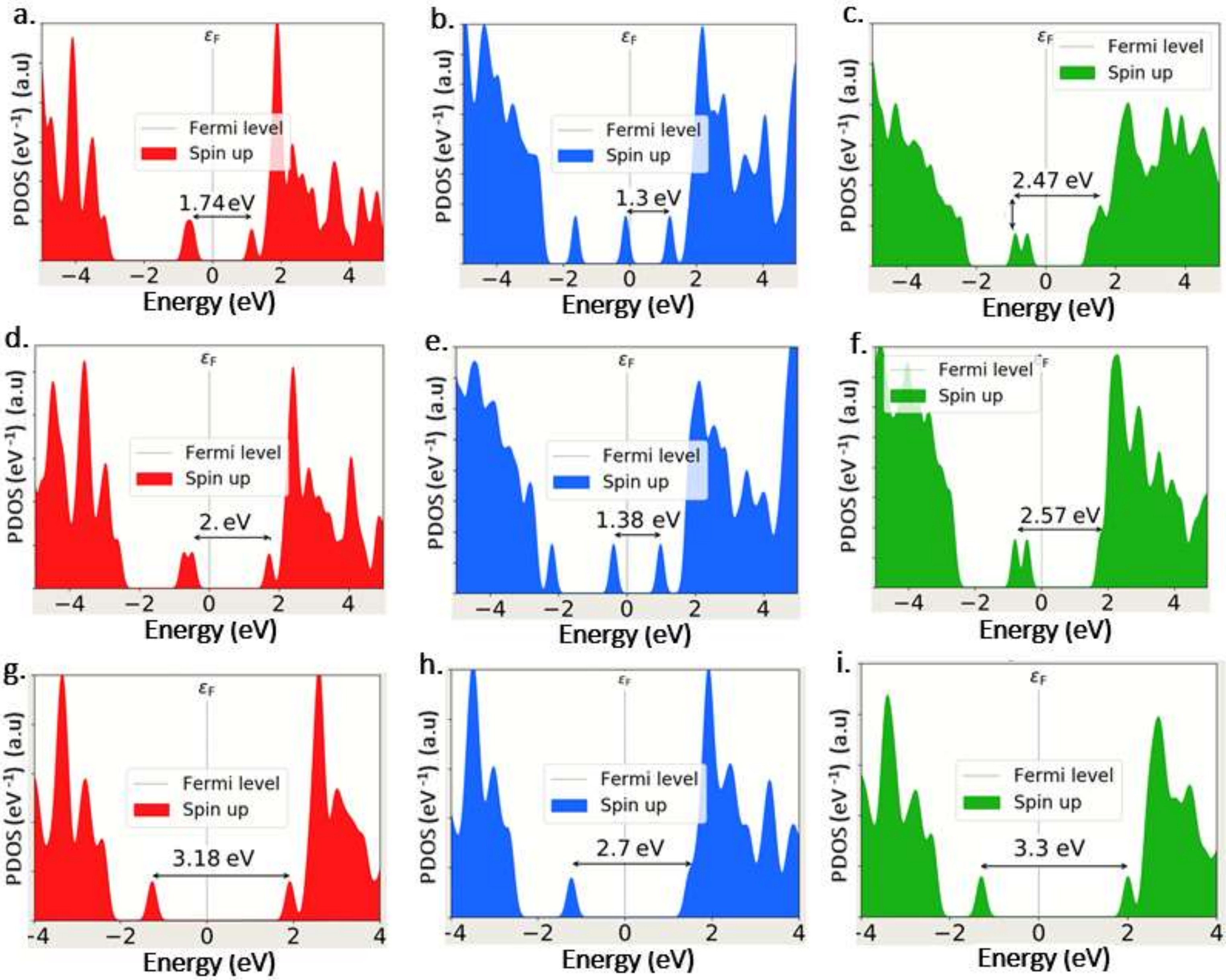

Figure S3: PDOS of C_BV_N, N_BV_N and boron dangling bonds quantum emitters under un-strained and other strained conditions. (a, d and g) Red coloured graphs are PDOS of quantum emitters (C_BV_N, N_BV_N and boron dangling bonds respectively) under un-strained conditions, whose electronic transition energy difference between inter energy states is consistent with ZPL energies and ensures the quantum emission. (b, e and h) Blue coloured graphs are PDOS of strained quantum emitters, whose emission is tuned towards lower energy region and tuned ZPL energies are consistent with inter-state energy differences, which confirms the quantum emission alter. (c, f and i) Green coloured graphs are PDOS of strained quantum emitters, whose emission is tuned towards higher energy region and tuned ZPL energies are consistent with inter-state energy differences, which confirms tuning of quantum emission. The DFT strain inducement calculations for obtaining this PDOS were performed using LCAO calculations as explained in methodology section.

`

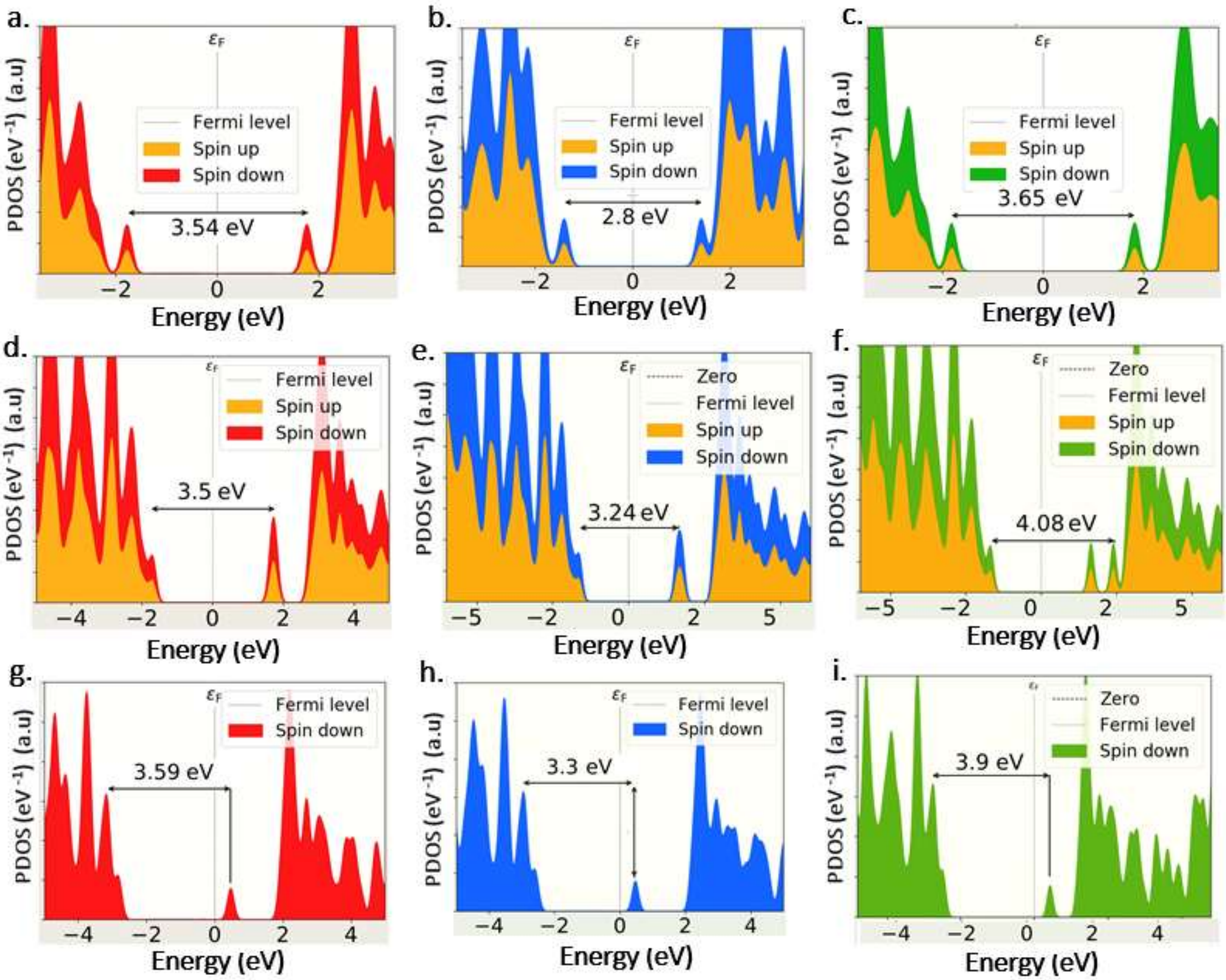

Figure S4: PDOS of C_BC_N, V_{BN} and V_N quantum emitters under un-strained and strained conditions. (a, d and g) Red coloured graphs are PDOS of quantum emitters (C_BC_N, V_{BN} and V_N respectively) under un-strained conditions, whose electronic transition energy difference between inter energy states is consistent with ZPL energies and ensures the quantum emission. (b, e and h) Blue coloured graphs are PDOS of strained quantum emitters, whose emission is tuned towards lower energy region and tuned ZPL energies are consistent with inter-state energy differences, which confirms the quantum emission alter. (c, f and i) Green coloured graphs are PDOS of strained quantum emitters, whose emission is tuned towards higher energy region and tuned ZPL energies are consistent with inter-state energy differences, which confirms tuning of quantum emission. The DFT strain inducement calculations for obtaining this PDOS were performed using LCAO calculations as explained in methodology section.

`

IV. Bond length alters of boron dangling bonds, N_BV_N and C_BV_N defects due to biaxial strain:

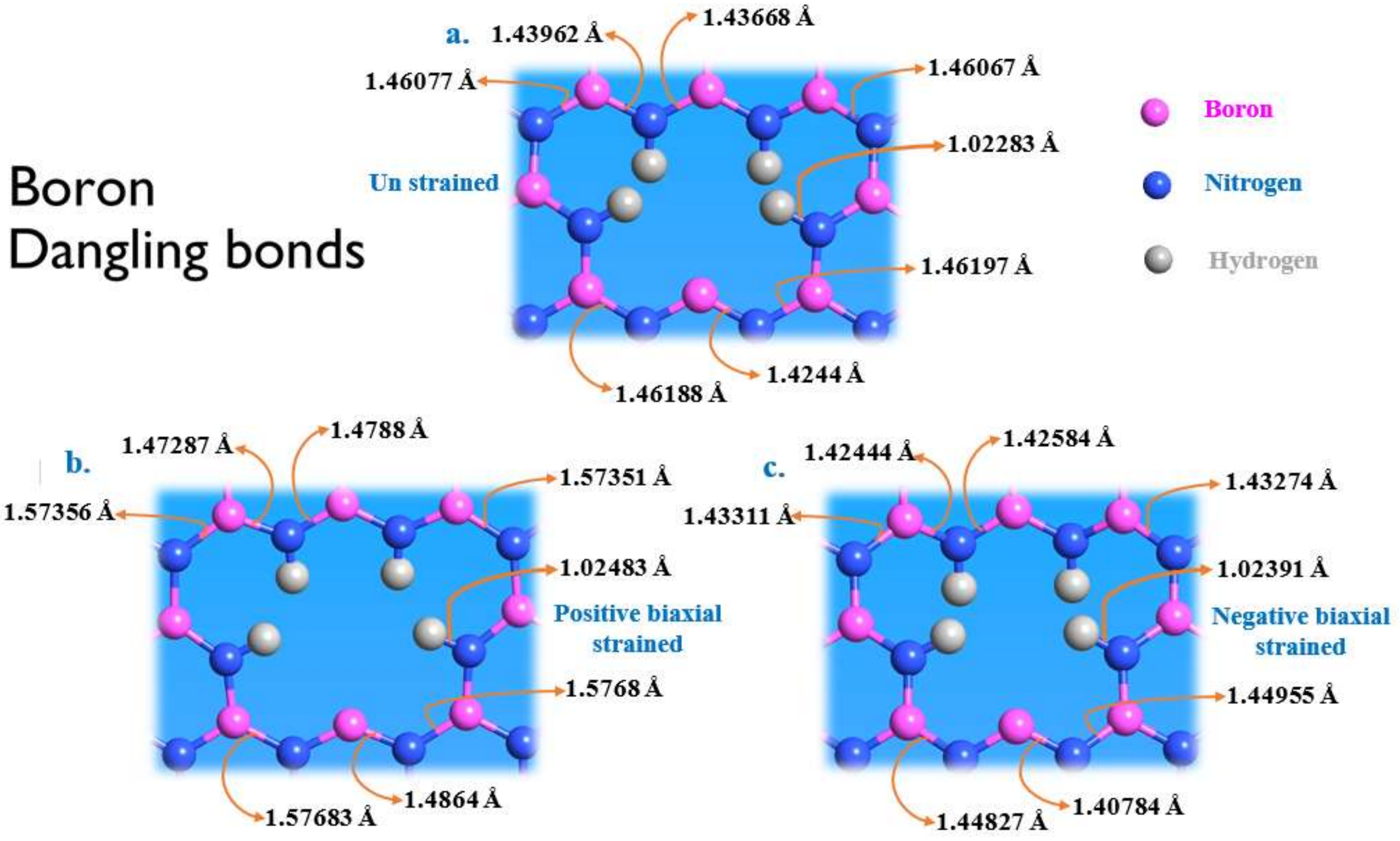

Figure S5: Schematic representation of bond length alters in boron dangling bonds due to biaxial strain inducement. (a) Optimized boron dangling bonds point defect and their corresponding bond lengths measured under no external inducement condition. (b and c) Boron dangling bonds point defects and their altered bond lengths measured under positive and negative biaxial strain inducements respectively. The bond lengths found incremented for positive biaxial strain and decremented for negative biaxial strain inducements, compared to bond lengths of point defect under no strain condition as shown in (a). The % of positive and negative strain applied to the boron dangling bonds defect was listed in Table 5.

`

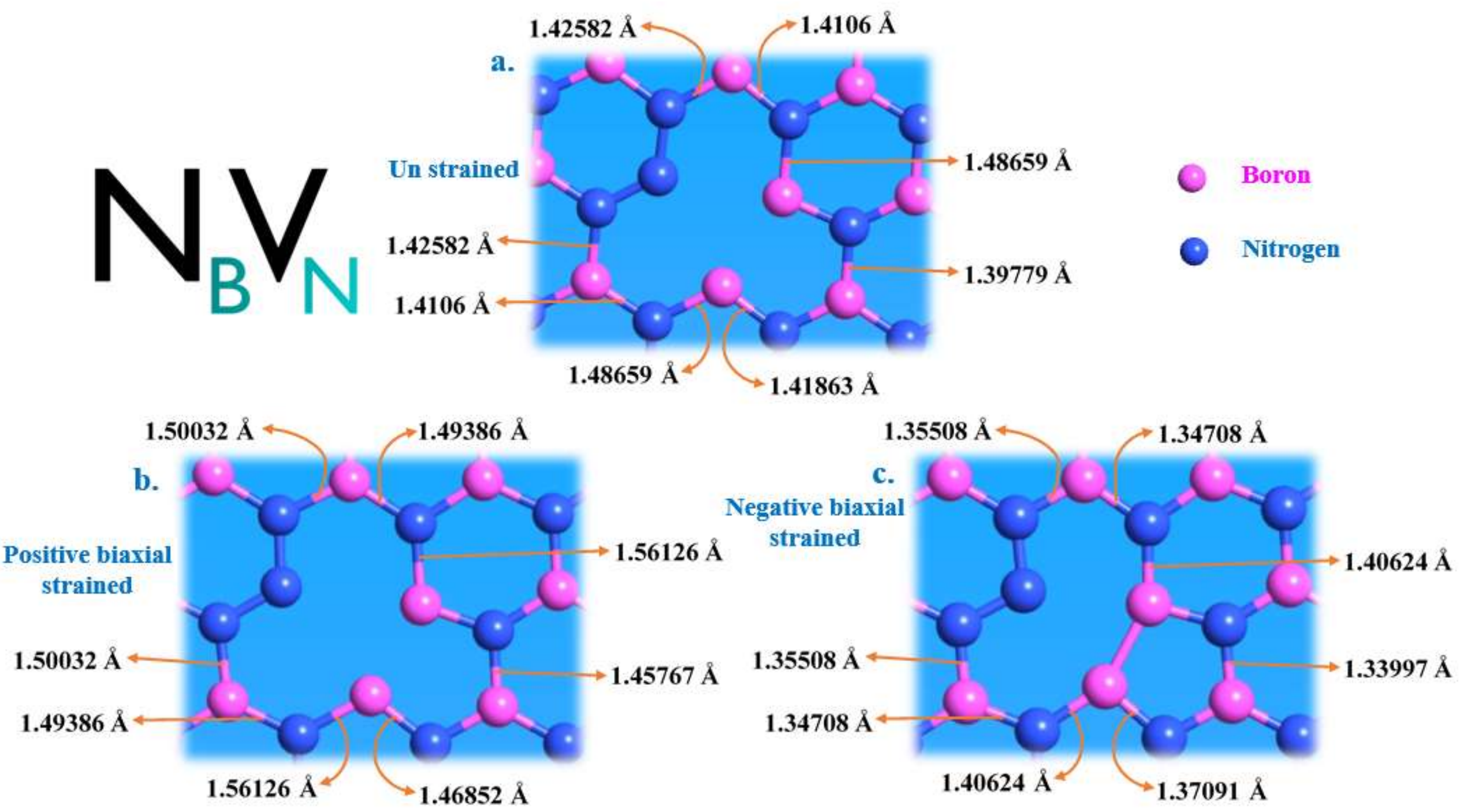

Figure S6: Schematic representation of bond length alters in N_BV_N defect due to biaxial strain inducement. (a) Optimized N_BV_N point defect and their corresponding bond lengths measured under no external inducement condition. (b and c) N_BV_N point defects and their altered bond lengths measured under positive and negative biaxial strain inducements respectively. The bond lengths found incremented for positive biaxial strain and decremented for negative biaxial strain inducements, compared to bond lengths of point defect under no strain condition as shown in (a). The % of positive and negative strain applied to the N_BV_N defect was listed in Table 5.

`

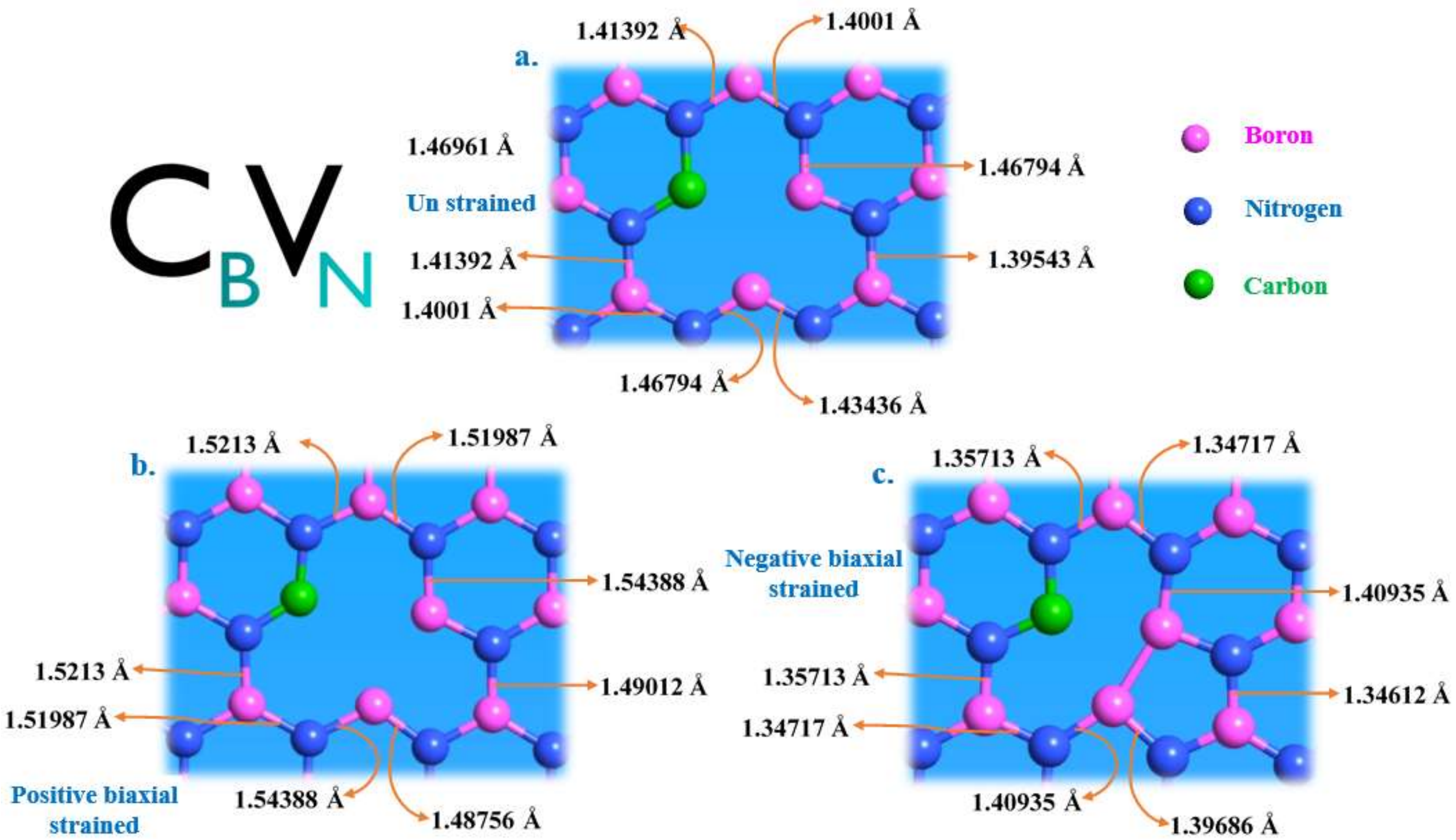

Figure S7: Schematic representation of bond length alters in C_BV_N defect due to biaxial strain inducement. (a) Optimized C_BV_N point defect and their corresponding bond lengths measured under no external inducement condition. (b and c) C_BV_N point defects and their altered bond lengths measured under positive and negative biaxial strain inducements respectively. The bond lengths found incremented for positive biaxial strain and decremented for negative biaxial strain inducements, compared to bond lengths of point defect under no strain condition as shown in (a). The % of positive and negative strain applied to the C_BV_N defect was listed in Table 5.

`

V. **Bond length alters of C_BV_N defects due to one-sided lateral and longitudinal strain inducement:**

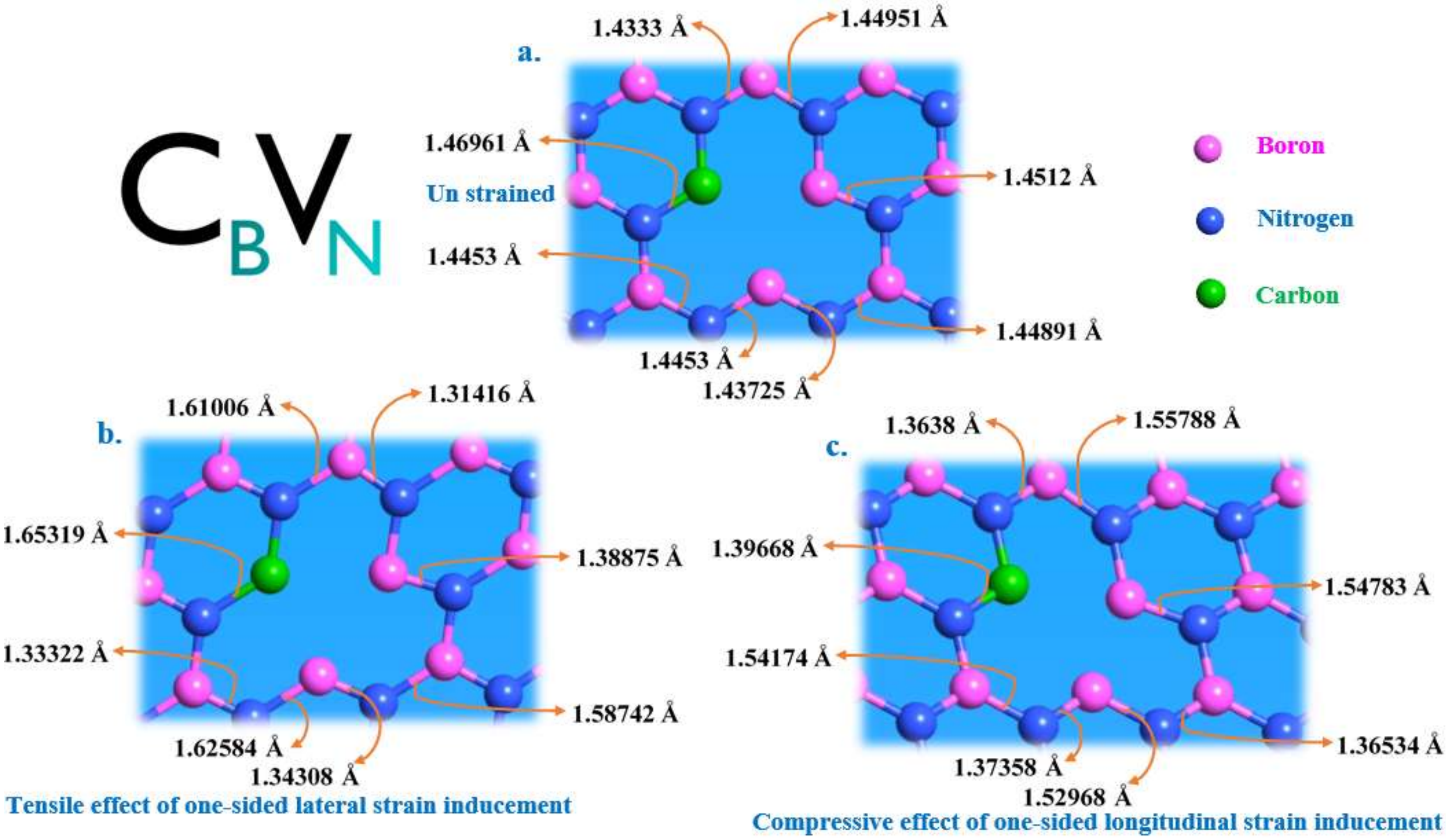

Figure S8: Schematic representation of bond length alters in C_BV_N defect due to one-sided lateral and longitudinal strain inducement. (a) Optimized C_BV_N point defect and their corresponding bond lengths measured under no external inducement condition. (b and c) C_BV_N point defect and their altered bond lengths measured under tensile effect of one-sided lateral strain and compressive effect of one-sided longitudinal strain inducements. Some of the bond lengths tends to increase and some of the bond lengths tends to decrease in both one-sided lateral and longitudinal strain inducements. This is due to the involvement of Poisson's ratio effect along with one-sided lateral and longitudinal strain inducements. The % of positive and negative strain applied to the C_BV_N defect was listed in Table 5.

VI. **Spin transitions preserved by luminescent point defects during inter energy state electronic transition, explored using DFT computations**

The information related to electronic spin of luminescent point defects was obtained by corresponding PDOS graphs of luminescent point defects, using DFT computations. If we observe all mono vacancy defects (V_B and V_N) were holding Spin-down (↓) transitions, mono vacancies with self and extrinsic interstitials

`

(N_BV_N, C_BV_N and V_BO_2) were revealing Spin-up (↑) transitions, dual vacancies (V_{BN}) and dual carbon replacement (C_BC_N) defects were preserving both Spin-up (↑) and Spin-down (↓) transitions. The Spin-up (↑) transition of N_BV_N defect was found to be consistent with former **[39]** VASP computations, performed using plane-wave basis sets.

Table ST1: Information related to type of spin transitions of point defects	
**Point defect**	**Type of spin can be preserved during inter energy state electronic transition**
V_BO_2	Spin-up (↑)
C_BV_N	
N_BV_N	
Boron dangling bonds	
V_B	Spin-down (↓)
V_N	
C_BC_N	Both Spin-up (↑) and Spin-down (↓)
V_{BN}	

References:

39. T. Tran, K. Bray, M. Ford, M. Toth and I. Aharonovich, "Quantum emission from hexagonal boron nitride monolayers", *Nature Nanotechnology*, vol. 11, no. 1, pp. 37-41, 2015.

`
